# Combined Allosteric Responses Explain the Bifurcation in Non-Linear Dynamics of ^15^N Root Fluxes Under Nutritional Steady-State Conditions for Nitrate

**DOI:** 10.3389/fpls.2020.01253

**Published:** 2020-08-28

**Authors:** Erwan Le Deunff, Patrick Beauclair, Julien Lecourt, Carole Deleu, Philippe Malagoli

**Affiliations:** ^1^ Normandie Université, UNICAEN, Caen, France; ^2^ Institute of Plant Sciences Paris Saclay (IPS2), CNRS, INRA, Université Paris-Sud, Université d’Evry, Université Paris-Saclay, Gif-sur-Yvette, France; ^3^ INRA Unité Expérimentale Fourrages Environnement Ruminants (FERLUS) et Système d’Observation et d’Expérimentation pour la Recherche en Environnement (SOERE) Les Verrines CS 80006, Lusignan, France; ^4^ NIAB EMR, Crop Science and Production Systems, East Malling, United Kingdom; ^5^ INRA—Agrocampus Ouest—Université de Rennes 1, UMR 1349 Institut de Génétique, Environnement et Protection des Plantes (IGEPP) Université de Rennes 1, Rennes, France; ^6^ Université Clermont Auvergne, INRA, PIAF, Clermont-Ferrand, France

**Keywords:** ion transport kinetics, nitrate uptake, N translocation, analysis of non-linear dynamic systems, irreversible thermodynamics

## Abstract

With regard to thermodynamics out of equilibrium, seedlings are open systems that dissipate energy towards their environment. Accordingly, under nutritional steady-state conditions, changes in external concentrations of one single ion provokes instability and reorganization in the metabolic and structure/architecture of the seedling that is more favorable to the fluxes of energy and matter. This reorganization is called a bifurcation and is described in mathematics as a non-linear dynamic system. In this study, we investigate the non-linear dynamics of ^15^N fluxes among cellular compartments of *B. napus* seedlings in response to a wide range of external NO3−15 concentrations (from 0.05 to 20 mM): this allows to determine whether any stationary states and bifurcations could be found. The biphasic behavior of the root NO3−15 uptake rate (*v_in_*) was explained by the combined cooperative properties between the *v_app_* (N uptake, storage and assimilation rate) and *v_out_* (N translocation rate) ^15^N fluxes that revealed a unique and stable stationary state around 0.28 mM nitrate. The disappearance of this stationary state around 0.5 mM external nitrate concentrations provokes a dramatic bifurcation in ^15^N flux pattern. This bifurcation in the *v_in_* and *v_out_* ^15^N fluxes fits better with the increase of *BnNPF6.3/NRT1.1* expression than *BnNRT2.1* nitrate transporter genes, confirming the allosteric property of the *BnNPF6/NRT1.1* transporter, as reported in the literature between low and high nitrate concentrations. Moreover, several statistically significant power-law equations were found between variations in the shoots tryptophan concentrations (i.e., IAA precursor) with changes in the *v_app_* and *v_out_* ^15^N fluxes as well as a synthetic parameter of plant N status estimated from the root/shoot ratio of total free amino acids concentrations. These relationships designate IAA as one of the major biological parameters related to metabolic and structural-morphological reorganization coupled with the N and water fluxes induced by nitrate. The results seriously challenge the scientific grounds of the concept of high- and low-affinity of nitrate transporters and are therefore discussed in terms of the ecological significance and physiological implications on the basis of recent agronomic, physiological and molecular data of the literature.

## Introduction

The integrated understanding of the nitrogen nutritional function at the whole plant level continues to remain elusive. In agro-systems nitrate is one of the most limiting macro-nutrients and its use as a fertilizer has increased steadily (Good et al., 2004; Garnett et al., 2009). Over the past two decades, the literature has clearly attempted to bridge the gap between the changes in short-term nitrate transporter activity and the physiological and molecular responses to nitrate to obtain an integrated view of plant nutrition (Goel et al., 2018; Vidal et al., 2020). The use of nitrate isotherm was the starting point for the identification and characterization of transporter genes in *Arabidopsis* and many other species (Tsay et al., 1993; Zhuo et al., 1999). An isotherm is a curve depicting the response of ion influx rate to changes in external concentrations of this ion at a given temperature, volume and pressure (Le Deunff et al., 2016a). The first isotherms were established about half a century ago for the potassium ion (K^+^) with the help of K^+^ (^42^K) or rubidium (^86^Rb) radioactive tracers in barley roots (Epstein et al., 1963). They revealed a biphasic K^+^ absorption pattern mainly explained by two distinct absorption mechanisms involving two different potassium transport systems (kinetic components of ion flux across the root): one acting at low external concentrations (< 0.5–1 mM) and the other operating for high external concentrations (> 1 mM). For ions, such as NO3−, K^+^, Cl^-^, and Ca^2+^ the underlying process responsible for the abrupt increase in ion influx rate between mechanism I and mechanism II beyond a threshold value around 0.5–1 mM has remained unexplained (Epstein et al., 1963; Thellier, 1973; Siddiqi et al., 1990). Understanding of this abrupt increase is critical for modern agriculture because this behavior is the basis for the use of fertilizers and the success of the green revolution for increasing the yield potential of modern crops (Le Deunff and Malagoli, 2014a). However, from the seminal works of Epstein and co-workers and their *Enzyme-substrate* interpretation of mechanisms I (renamed HATS for Low-Affinity Transport System) and II (renamed LATS for High-Affinity Transport System) of ion isotherms, a dynamic explanation about the abrupt transition between these two mechanisms has not been found yet (Le Deunff and Malagoli, 2014a).

**Figure 1:**
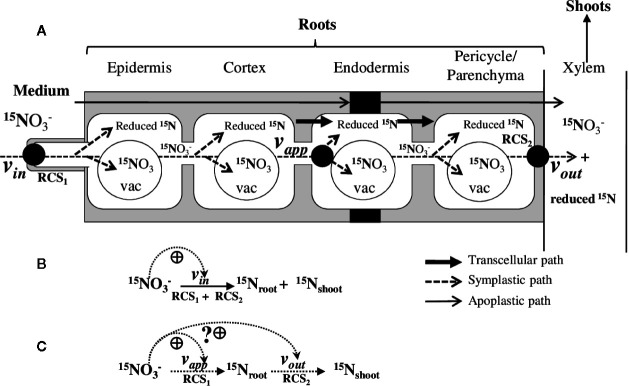
Representative model of the NO−315 nitrate absorption into the roots and ^15^N translocation to the shoots. **(A)** Three compartments model deduced from the ^15^N analyses of *B. napus* seedlings growing under nutritional steady-state conditions where external ${\mathrm{NO}}_{3}^{-}$ concentration is the only changing variable. RCS1 and RCS2 correspond to the Root Catalytic Structures involved in net nitrate uptake at the root epidermis level and net ^15^N translocation to the shoots from the root vasculature tissues, respectively. Therefore, *v_in_* and *v_out_*represent the summed activities of the different transporters (e.g., nitrate and amino acids influx and efflux transporters) that are involved in NO3−15 uptake, (*v_in_*), storage, and assimilation of NO3−15 and ^15^N–reduced forms (*v_app_*) and ^15^N translocation to the shoots of NO3−15 and ^15^N–reduced forms (*v_out_*). The model does not take into account for the compartmentation of NO3−15 and ^15^N-reduced forms in the roots and ^15^N reduced forms cycling between the root and shoot. Both mechanisms were supposed to be stable under steady-state nutritional conditions. **(B)** Classic model used from the ^15^N tracers to establish the isotherm of nitrate at the root plasma membrane. **(C)** Model used in this study to interpret ^15^N fluxes along the symplastic path from the uptake rate of nitrate at epidermis level to loading rate of nitrate into the xylem. Dashed arrows indicate the ante-activation associated with the nitrate-induced transporters activity and transcription at the epidermis and root vascular tissues.

In the case of nitrate, the molecular characterization of the nitrate transporters involved in the mechanisms I and II did not elucidate the shape of the isotherm response curve (Wen and Kaiser, 2018). Because the nitrate transporters are the first involved in the intake of nitrate into the root epidermal cells, it was tempting to assign them a signaling role as a transporter and receptor (i.e., transceptor) in an attempt to explain the biphasic pattern of nitrate absorption and the changes in metabolism and growth in response to nitrate availability (Bouguyon et al., 2012). Accordingly, the signaling role of some transporters such as *NPF6.3* (also known as *NRT1.1* and *CHL1*) belonging to the Nitrate transporter 1/peptide transporter family (NPF) has been investigated in three different signaling pathways. These interpretations propose explanations for either the biphasic response of *NRT2.1* nitrate transporter gene expression (Hu et al., 2008; Ho et al., 2009), the priming effect of nitrate on nitrogen metabolism (Hu et al., 2008), and the root foraging behavior in soil nitrate rich-patch (Remans et al., 2006b). In addition, the wide range of substrates (K^+^, Cl^-^, $NO_{3}^{-}$, peptides, amino acids, auxin, ABA,…) for the *NPF6.3*-like transport proteins of the NPF family highlights the variability of their functional capabilities and questions their major importance for ion fluxes and plant growth in response to nitrate (Wen et al., 2017; Wen and Kaiser, 2018). In fact, these top-down (from isotherm to transporter) and bottom-up (from transporter to isotherm) approaches are often discordant because molecular physiologists have to cope with emergent functional and morphological behaviors to nitrate that cannot be deduced uniquely by the sum of corresponding sub-systems or gene regulation identified from transcriptomic studies or approaches of system biology (Gallagher and Appenzeller, 1999; Alekhina et al., 2000; Ricard, 2006; Horaruang et al., 2020). Likewise, ion isotherms represent an integrated view of ion uptake which does not reflect the underlying dynamic fluxes of nitrate that depends on ion transporter activity throughout the root symplastic pathway (**Figure 1A**). Therefore, this apparent and static approach hinders a more explanatory and dynamic integrated view of the plant nutritional system.

From a dynamic point of view, nitrate uptake cannot be reduced to the mere acquisition by the root’s epidermis (Alekhina et al., 2000; Horaruang et al., 2020). Indeed, this represents an over-simplification of the symplastic pathway of root nitrate transport (**Figure 1A**) which also involves the nitrate assimilation and accumulation rates into the vacuole and plasts as well as nitrate loading rate into the xylem (Scheible et al., 1997; Britto and Kronzucker, 2001b; Britto and Kronzucker, 2003; Le Deunff et al., 2016a). Additionally, the nitrate uptake process and regulation at the root level is not only due to one type of nitrate transporters family, but summed activities of multiple carrier families (i.e., *NRT2*, *NPF*, *CLC*). These are arranged in series and/or parallel along the epidermis of the root segments and in the different root cell layers and cellular compartments (i.e., tonoplast and apoplast) along the root’s radius that form highly interacting catalytic structures for balancing electric charges. In simpler terms, the behavior and interactions of multiple ion transporters along the symplastic pathway are aggregated for a single ion transport into an emerging mathematical function (linear, sigmoidal, or hyperbolic) corresponding to the isotherms of this ion.

Moreover, despite their apparent simplicity in construct, interpretation of ion nutrient isotherms is not as straightforward. For example, the classical enzyme-substrate interpretation of the ionic isotherms is inevitably weakened by the debate of enzymologists between equilibrium conditions and stationary conditions (Cornish-Bawden et al., 2005). Indeed, a thermodynamic system is at equilibrium if the properties of the system are independent of time and no flow of matter or energy passes through the system. In this case, the laws of thermodynamics remain applicable to systems whose macroscopic parameters vary only very slowly compared to the time scale of the experiment. A thermodynamic system is in a stationary state if the system is crossed by a flow of matter and/or energy but the properties of the system do not change over time. In other words, the flows of matter and energy are stable and relaxed. Therefore, when physiologists build ion isotherms, they have to account for the experimental conditions used such as the rinsing conditions and ion acclimation of the plant roots to minimize disturbance of thermodynamic conditions during influx rate measurements (Britto and Kronzucker, 2001a; Le Deunff et al., 2016a). When interpreting isotherms, physiologists face the veracity of the simplifying assumptions used to coherently interpret the measurements of ion influx rate. An alternative approach consists of considering that nitrate uptake isotherms are always obtained out of the equilibrium condition, because plants are dissipative structures that always exchange matter and energy with the environment, even over a short period of time and under low concentrations of substrate. Therefore, the analysis of dynamic NO3−15 or NO3−13 fluxes are only possible under nutritional steady-state conditions. As indicated above, the steady-state nutritional conditions are defined as a state of the plant system in which a flow of matter and/or energy crosses the system (i.e., plasma membranes of the root cells) but for which the properties of the system do not vary over time. Under these conditions the system is relaxed and its inputs are no longer modified.

A first method under nutritional steady-state conditions consists of using a (Sub)-Compartmental Analysis of NO3−13 Tracer Exchange (CATE or SCATE) to estimate the half-lives (t_0.5_) for NO3−13 residence within the cytosolic compartment (Walker and Pitman, 1976; Britto and Kronzucker, 2001b; Britto and Kronzucker, 2003). Thus, CATE or SCATE analysis has demonstrated existence of a fluxes coordination between efflux from cytosol to the cell wall (Φco), fluxes across the tonoplast (ϕcv and ϕvc), flux from cytosol to the xylem (ϕcx), as well as fluxes required for organic synthesis (Britto and Kronzucker, 2001b; Britto and Kronzucker, 2003). The theoretical model of nitrate uptake and coordinated cellular fluxes deduced from CATE is composed of four compartments: external medium (apoplast), cytosol, vacuole, and xylem. This model matches better with the location of the different types of nitrate transporters identified by recent molecular data and located from root epidermis to the vascular tissues (Pitman, 1976; Le Deunff and Malagoli, 2014b).

A second method consists of the analysis of non-linear dynamics of ^15^N or ^13^N fluxes in seedlings under nutritional steady-state conditions. Under these conditions, changes in external concentrations of one single ion such as nitrate leads to instability, then reorganization in the metabolic pathways and root structure/architecture of seedlings, favoring fluxes of energy and matter under such perturbation (Remans et al., 2006a; Le Deunff et al., 2016b). The mathematical study of bifurcations falls into non-linear dynamic systems analyses (Laurent et al., 2005; Laurent, 2013). If considering plants as an open system that dissipates energy and exchange matter, then plants must exhibit expected properties of a dissipative structure in response to increasing external nitrate concentrations (Barbacci et al., 2015). This means that the emerging properties are part of a more general framework and provides a better understanding of the integrated view of emergent plant nutritional responses (for details and explanations see Barbacci et al., 2015).

To our knowledge, this second thermodynamic method has not been used to date to analyze plant response to nutrient-ion availability such as nitrate. This study presents a re-examination of the ^15^N fluxes in seedlings of *B. napus* growing under nutritional steady-state conditions in agar plates over a wide range of external nitrate concentrations (from 0.05 to 20 mM) in order to determine whether stationary states and bifurcations in the dynamic fluxes of ^15^N may be found. Our analysis revealed the presence of a single stationary state, locally stable, whose disappearance conducts to a bifurcation in ^15^N translocation rate to the shoots in response to increase in nitrate availability. We also investigated whether major morphological, biochemical and transcriptional changes of nitrate transporters *NPF6.3/NRT1.1* and *NRT2.1* were associated with this bifurcation.

## Materials and Method

### Plants Culture

The *Brassica napus* L. seeds of winter oil seed rape (*cv* Capitol) were treated for germination according to Leblanc et al. (2008). After 48 h sowing in the dark at room temperature, four seedlings were selected by their radicle length (5–6 mm), and were transferred to new Petri dishes (12 x12 cm) filled with 50 mL of solidified agar culture medium. Basic medium contained 0.4 mM KH_2_PO_4_, 0.15 mM K_2_HPO_4_, 1 mM K_2_SO_4_, 0.5 mM MgSO_4_, 3 mM CaCl_2_, 0.2 mM Fe-Na EDTA of macronutrients and 14 μM H_3_B0_3_, 5 μM MnSO_4_, 3μM ZnSO_4_, 0.7 μM CuSO_4_, 0.7 μM (NH_4_)_6_ Mo_7_O_24_, and 0.1 μM CoCl_2_ of micro-nutrients and was solidified with 0.8% (W/V) agar (Sigma A-7002), pH 6.75. This basic medium was supplemented with KNO_3_ as the sole nitrogen source at the concentrations indicated for each individual experiment (from 0.05 to 20 mM). Then, the Petri dishes were half sealed with adhesive tape. The dishes were placed vertically in a growth chamber at 22°C under a 16/8 light/dark regimen with a photosynthetic photon flux density of 250 μmol m^-2^ s^-1^.

### Physiological Plant Experiments

#### Experiment 1, Seedlings Responses to Nitrate Availability

The net uptake of KNO_3_ was obtained by homogeneous labeling with ${\text{K}}^{15}\text{N}{\text{O}}_{3}$ (atom % ^15^N: 1%) by using serial sampling at time points of 24 h, 48 h, 72 h, 96 h, and 120 h after nitrate treatments. The KNO_3_ concentration was the only changing variable and varied as much as 400-fold from 0.05 to 20 mM (i.e., 0.05; 0.2; 0.5; 1; 5; 10 and 20 mM). All the measurements were done on four seedlings mixed together provided by four agar plates carrying four seedlings (i.e., four replicates, N=4). For each sampling time, the roots and shoots of nitrate-treated seedlings were separated. Then, plants organs were weighed (fresh weight) and dried (dry weight) in an oven for 72 h at 60°C. Before total N and ^15^N analyses, dry tissues were ground for 2 min to a fine powder with 5 mm diameter stainless beads in an oscillating grinder (Retsh mixer mill, MM301). ^15^N accumulated in roots and cotyledons were then analyzed by elemental analyzer (analyzer EA 3 000, Eurovector, Milan, Italy) coupled with an isotope ratio mass spectrometry (IRMS, isoprime X, GV instrument).

#### Experiment 2, Seedlings Responses to Nitrate Availability With an Excised-Cotyledon

The experiment 2 was performed under the same conditions as experiment 1, except that the net uptake rate and accumulation of ${\text{K}}^{15}\text{N}{\text{O}}_{3}$ were obtained over 120 h of nitrate treatment and the KNO_3_ concentration varied from 0.2 to 20 mM (i.e., 0.2; 0.5; 1; 2.5; 5; 10; and 20 mM). The seedlings were divided into two sets. For the first set of seedlings, a single cotyledon was removed with a pair of scissors (ablation treatment) when the seedlings started their autotrophic growth phase after the greening of cotyledons. The other set of seedlings was kept as control.

#### Experiment 3, Seedlings Responses to Glutamate Treatment in the Presence of 1 and 5 mM KNO_3_

Basic medium of Petri dishes in control treatments were supplemented with KNO_3_ as the sole N source at the concentrations indicated for each individual experiment (from 1 to 15 mM). For measurements with Glutamate (Glu) treatments, the basic medium was supplemented by increasing Glu concentrations (Sigma G-1501) from 0.25 to 10 mM (i.e., 0.25; 0.5; 1; 2.5; 5; and 10 mM) with 1 or 5 mM KNO_3_. Therefore, in control and Glu treatments, the seedlings were fed with a constant molarity in total N supply.

### Morphometric Analyses

The root hair length along the primary root axis in the basal part (BLEH = Basal Length of Epidermal root Hair cell) of the neo-elongated primary root was measured on treated seedlings with the different concentrations of KNO_3_ between 48 and 72 h of treatment, as described by Leblanc et al., 2008. Only six seedlings showing the elongation mean value were chosen for these measurements. Palisade cells of the fully expanded cotyledon after 72 h of nitrate treatments were fixed and cleared according to the methods of Young et al. (1979). The root hair length and palisade cell diameters were then measured respectively under a light microscope (Leica CME, Buffalo, New York USA) at 40 x magnification equipped with a camera (JVC, TK-C1481BEG, Korea) and video imaging system (Sony Trinitron, PVM-20L1, Japan) and a Nomarski microscope Nikon (Optiphot 2).

### Stomatal Conductance and Photosynthesis Measurements

After 120 h of growth on agar plates, an independent set of seedlings treated with different concentrations of nitrate (i.e., 0.05; 0.2; 0.5; 0.75; 1; 2; 3; 4; 5; 10; 15; and 20 mM) were used for measurements of the cotyledon conductance (*g_s_*) and gas fluxes. Cotyledons of six *B. napus* seedlings per nitrate concentration were measured using a Li-COR^®^ 6400 system (Inc., Lincoln, NE) and *Arabidopsis* 6400-15 extended reach 1cm chamber. The conditions in the chamber during the measurement were as follows: a flow rate of 120 to 200 μmol s^-1^, PAR of 200 μmol m^-2^ s^-1^, relative humidity of 70%, and an air temperature of 24°C. Photosynthesis values were optimized to take into account the changing CO_2_ concentrations in the chamber during the experiment by measuring the concentration of CO_2_ entering in the empty chamber between each treatment.

### RNA Isolation and Quantitative RT-PCR Analysis

The total RNA from four treated seedlings was extracted from 200–400 mg of fresh root tissues frozen in liquid nitrogen and ground for 1.5 min with 4 mm diameter inox beads in an oscillating grinder (Retsh mixer mill, MM301). Total RNA was then extracted from the fresh frozen powder using the RNA easy plant mini kit (Qiagen) and quantified before RT-PCR analyses by spectrophotometer (Biophotometer, Eppendorf). The quantitative RT-PCR was performed according to Leblanc et al. (2008) with a PTC-200 DNA Engine cycler (Bio-rad). Expression levels of *BnNRT2.1* and *NPF6.3/BnNRT1.1* nitrate transporters genes were normalized to the expression level of the *18S* house-keeping gene. The primers used to amplify the specific sequences after RT were: for ribosomal *18S* gene (F, 5’-cgg ata acc gta gta att cta g; R, 5’-gta ctc att cca att acc aga c), for *BnNRT2.1* (F, 5’-tgg tgg aat agg cgg ctc gag ttg and R, 5’-gta tac gtt ttg ggt cat tgc cat; AJ293028) and for *NPF6.3/bnNRT1.1* gene *(F, 5’*-atg gtaacc gaa gtg cct tg; R, 5’-tga ttc cag ctg ttg aag c, AJ278966).

### Amino Acid Profiling by UPLC Analyses

Amino acid profiling was performed on the freeze-dried tissues of the shoot and root after homogenization with 4 mm diameter stainless steel beads in an oscillating grinder for 1 min at 30/s frequency (Retsh mixer mill, MM301). 10 mg of the resulting tissue dry powder was used for methanol-chloroform-water-based extraction according to Renault et al. (2010). After extraction, a 5 μl aliquot of the resulting suspended dried extracts with 600 μl of ultra-pure water was used for derivatization according to the AccQTag Ultra Derivatization Kit Protocol (Waters Corp.), and then the derivatized amino acids were analyzed by using an ACQUITY UPLC system (water Corp.). The content of individual amino acids was expressed in μmoles per g of dry weight tissue by reference to the DL-3-aminobutyric acid (BABA) taken as an internal standard and to external calibration curves of amino acids.

### Analysis of Non-Linear Dynamics of ^15^N Flows

#### Definition of Nutritional Steady-State Conditions

The steady-state nutritional conditions for absorption and growth are defined as the situation where the net fluxes into and out of the root compartments are constant as are the external nitrate concentrations used. The nutritional steady-state conditions are reached when the system is relaxed and the root ^15^N inflows (*v_in_* and* v_app_*) and outflow (*v_out_*) to the shoots are no longer modified (see below paragraph for the explanation on *v_in_*, *v_app_*,**and *v_out_*). In seedling experiments on agar plates, the non–linear dynamics of the ^15^N fluxes in the roots and shoots can only be compared from 48 h after sowing when the seedlings start their autotrophic growth phase. As shown in **Figure S1**, the flow dynamics of ^15^N are similar after 120 h, 96 h, and 72 h of labeling, which indicates that the system is under ^15^N isotopic steady-state. Likewise, the day-night cycle variations of ^15^N fluxes were also very similar during the successive day-night cycles between 48–72 h, 72–96 h, and 96–120 h (**Figure S2**). Similar patterns of dynamic fluxes during the time-course of experiment indicates that recycling of ^15^N-reduced forms and nitrate translocation remained stable under nutritional steady-state conditions between the successive day-night cycles. Therefore, estimated of the *v_app_* and *v_out_* ^15^N fluxes were made assuming recycling of ^15^N remains constant between shoots and roots during the nitrate treatments.

### Determination of Stationary States Under Nutritional Steady-State Conditions

Here, the seedling is considered as an open system (**Figure 1A**) with only one changing variable (i.e., the substrate concentration *S_j_*) where *v_in_* is the velocity of the substrate *S_j_* into the roots (i.e., net NO3−15 uptake rate) and *v_out_* is the velocity of net *S_j_* elimination rate from the roots (e.g., net translocation rate of NO3−15 + ^15^N reduced forms). In fact, *v*_in_ corresponds to summed activities (= *v*_in1_ *+ …+ v*_inn_) of different nitrate transporters involved in transport across the plasma membrane of the epidermis root cell layer with velocities *v_in1_*, …, *v*_inn_. Likewise, *v_out_* results from the summed activity of transporters (*v*_out1_+ …+*v*_outn_) that are involved in loading of ^15^N (*i.e.* NO3−15+15N reduced forms) into xylem (**Figure 1A**). Since NO3−15 taken up by the root epidermis can be stored under nitrate or ^15^N-reduced forms in vacuoles and cytosol of the root cells, there is a secondary pathway to remove ^15^N from the root compartment (**Figure 1A**). This secondary path also takes into account the ^15^N-reduced forms such as amino acid cycling between the roots and shoots. Therefore, this secondary path is defined as an apparent uptake of NO3−15 absorption by the roots called apparent velocity (*v_app_*), which is obtained by subtracting the amounts of ^15^N present in the shoots from amounts of ^15^N in shoots + roots tissues. The dynamic of ^15^N fluxes without taking into account the compartmentalization of the ^15^N forms can be summarized in more a simplified schematic representation (**Figure 1B**). This formalization allows the determination of non-linear dynamics in ^15^N fluxes within the roots induced by changes in external nitrate concentrations. Accordingly, comparison of dynamic evolutions of *v_app_*and* v_out_* make it possible to graphically determine stationary states under steady-state nutritional conditions when:

$$d{[}^{15}N]/dt=v_{app}-v_{out}=0$$

The next step consists of determining graphically or mathematically if the stationary states are locally stable or unstable. Indeed, disappearance of a locally stable stationary state, or its transformation into a locally unstable stationary state, corresponds to a bifurcation.

### Mathematical Determination of the Stability of the Stationary States

The dynamic behavior of a system with a single independent changing variable (here $X={\left[NO_{3}^{-}\right]}_{ext}$), is given by a specific integrated solution from the non-linear differential equation:

$$X(t)=dX/dt=f(X,(p_{k}))$$

where the *p_k_* are the parameters of the system. The specific values that satisfy:

$$dX/dt=f(X,(p_{k}))=0\;or\;f(X)=0$$

correspond to stationary states and denoted $X^{*}={\left[NO_{3}^{-}\right]}_{ext}^{*}$. In fact, these stationary states are the roots of the non-linear equation, in our case given by:

f*(X)=d[N15]/dt=vapp−vout=0

In accordance with the mathematical method presented in appendix, the stability of a stationary state is tested by application of small perturbations *ΔX_0_*around the stationary state X^*^ (denoted $X(0)=X^{*}+\Delta X_{0}$) and depends on the sign of the derivative *f(X)* function (denoted *f’(X)*) calculated for $X^{*}={\left[NO^{\_}\right]}_{ext}^{*}$ when f*(X)=d[N15]/dt=vapp−vout=0. The linearization of the derivative function *f’(X)* around X^*^ when f ^*^(X) = 0 is obtained by a Taylor’s series development limited to the first order of *f(X)* (see appendix).

## Results

### In Steady-State Nutritional Conditions, Seedlings Exchange Matter and Energy From Low Levels of External Nitrate Concentration and Are Out of Equilibrium

**Figure 2:**
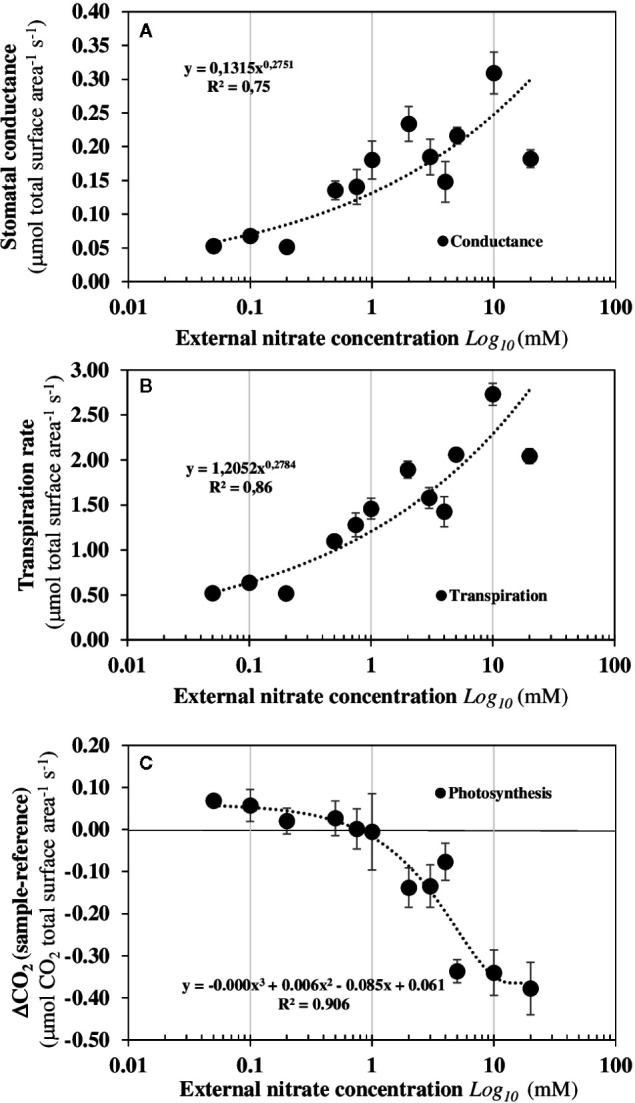
Under nutritional steady-state conditions, seedlings are dissipative structures out of equilibrium that exchange energy and matter with the environment. Changes in the stomatal conductance **(A)**, transpiration rate **(B)**, and photosynthesis rate **(C)** in response to nitrate availability. Values are the average ± SE of six individual seedlings measured after 120 h of treatment with varying external nitrate concentrations for 5 days in agar plate.

To verify that *B. napus* seedlings growing in steady-state nutritional conditions are open systems that exchange energy and matter with the environment, measurements of photosynthesis and respiration of the seedlings after 120 h of nitrate treatment were monitored in response to nitrate with a Licor-system 6 400 on individual seedlings (**Figure 2**). Increasing nitrate external concentrations induced a significant increase in shoot conductance at 0.5 mM external nitrate concentration (**Figure 2A**) that was associated with a significant increase in the shoot transpiration (**Figure 2B**). However, significant net carbon assimilation due to photosynthesis was only observed beyond 1 mM external nitrate concentration and increased for external nitrate concentration up to 20 mM (**Figure 2C**). Overall, these results indicate that under nutritional steady-state conditions used, seedlings exchange significant amounts of water, absorb nitrogen and fix carbon from their environment from low levels of external nitrate concentration.

### Nitrate Availability Causes a Break in Symmetry at the Cellular Level Along the Apical to Basal Axis

**Figure 3:**
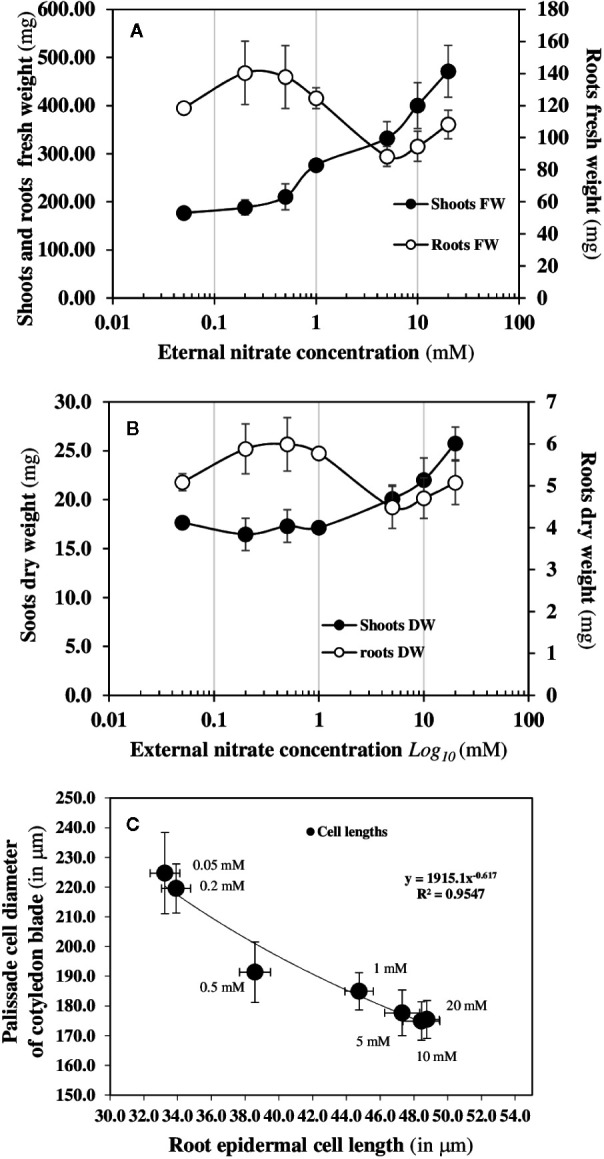
Structural reorganisation at cellular and organ levels induced by nitrate availability. Changes in the shoots and roots fresh **(A)** and dry **(B)** biomass after 120 h of nitrate treatment of *B. napus* growing on agar plates with increasing external nitrate concentrations from 0.05 to 20 mM. **(C)** Relationship between the isodiametric expansion of palisade cells in the central region of the cotyledon blade and the elongation of the “basal length of epidermal root hair cell in response to nitrate availability after 24 h of growth between 48 to 72 h nitrate treatment of *B. napus* seedlings in response to nitrate availability. Values are means ± SD of 17–23 root cells and 36 shoot palisade cells provided by 6 different seedlings (adapted from Le Ny et al., 2013).

The mirrored evolutions of the fresh and dry weights of the roots and shoots in response to increased external nitrate concentrations confirm that nitrate induces a structural reorganization (**Figures 3A, B**). This structural reorganization results in a break in symmetry between the roots and shoots growth along the apical-basal axis formed by the primary root and hypocotyl. The increase in shoot fresh weight occurs at 0.5 mM and is associated with a significant increase in the cotyledons conductance and transpiration which branch out between 0.2 and 0.5 mM (**Figures 2A, B**). However, the increase in the shoot dry weight occurs at 1 mM and is explained by a net carbon accumulation that only starts at 1 mM (**Figure 2C**). Because changes in the water cell volume between the shoots and roots occurs before the expansion and carbon accumulation in the cotyledons, the cell elongation in the primary root and cell expansion in the center of cotyledons’ blades were measured by light and Nomarski microscopy (**Figure 3C**). A significant negative correlation was found between the reduction in elongation of mature epidermal root hair cells along the primary root axis and the enlargement of palisadic cells in the central region of cotyledon blade in response to nitrate availability from 0.05 to 20 mM (**Figure 3C**). This result confirms that the break in symmetry at the cellular level along the apical-basal axis starts between 0.2 and 0.5 mM of external nitrate concentrations. This is much earlier than the organ expansion and carbon accumulation in the cotyledons. The obvious follow-on question is: is the nitrate-induced break of symmetry in cell growth associated with a biphasic behavior in the non-linear dynamic of ^15^N fluxes, as observed for the nitrate isotherms?

### In Steady-State Nutritional Conditions, Velocity of NO3−15 Fluxes Shows a Biphasic Behavior

**Figure 4:**
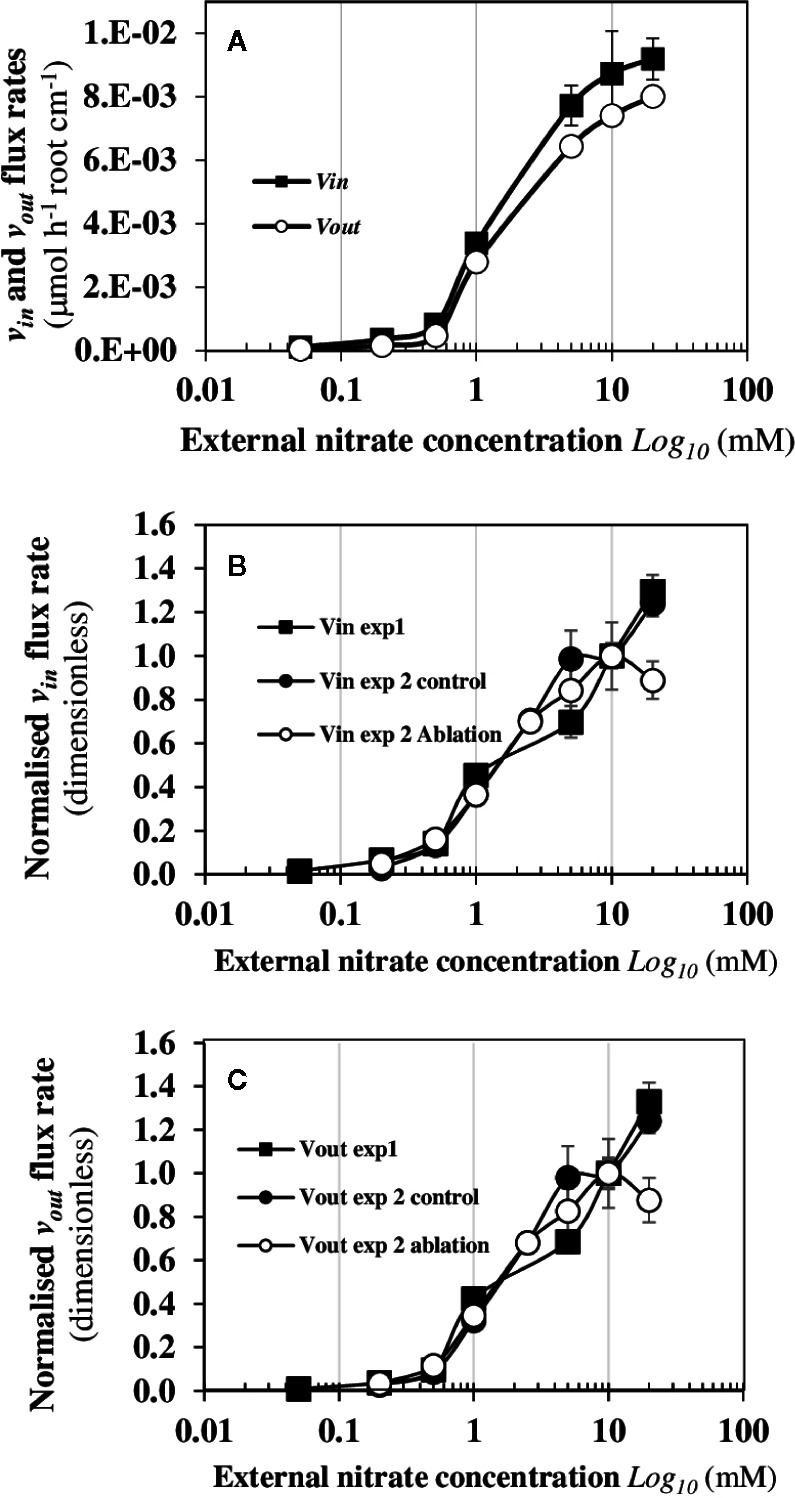
Biphasic behaviour of *v_in_*and *v_out_* ^15^N dynamic fluxes from *B. napus* seedlings in response to nitrate availability. **(A)** The *v_in_* and *v_out_* fluxes of *B. napus* seedlings were measured after 120 h treatment with a NO3−15 labeling (atom % ^15^N: 1%). **(B)** Comparison of the *v_in_* ^15^N flux between two independent experiments (exp1 et 2, see Mat and Meth) after 120 h treatment with of a ^15^N labeling. **(C)** Comparison of *v_out_* flux rates between two experiments (exp1 et 2) after 120 h treatment with a NO3−15 labeling. In the experiment 1, *B. napus* seedlings were intact whereas in the experiment 2, one set of the seedlings were intact (control) and the other set was excised for a single cotyledon (ablation). Seedlings were growing in nutritional steady-state conditions where the external $\text{N}{\text{O}}_{3}^{-}$ concentration is the only changing variable and varied from 0.05 to 20 mM. Values are means ± SD of four repeats of four seedlings each.

The dynamic patterns of ^15^N fluxes show that *v_in_* and *v_out_* are strictly parallel during increasing external nitrate concentrations (**Figure 4A**). The velocity *v_in_*corresponds to the total net uptake of ^15^N by the seedling roots whereas *v_out_* flux is the net ^15^N translocation rate to the shoots. The overall trend in *v_in_* and *v_out_* is associated with a biphasic behavior in the ^15^N translocation fluxes to the shoots with a dramatic increase of the N fluxes that occurs around 0.5 mM external nitrate concentration (**Figure 4A**). This highlights important inputs of matter *via* water fluxes and carbon fixation (**Figure 2**). At a morphological level, this biphasic behavior of ^15^N flux was associated with strictly opposite responses between enlargement of palisade cells in the central cotyledon blade and reduction in elongation of epidermal root hair cells along the primary root axis in response to nitrate (**Figure 3**, Le Ny et al., 2013). The strict parallelism between *v_in_* and *v_out_* ^15^N fluxes indicates a simultaneity of the ^15^N fluxes between the epidermis and the vascular tissues, which requires a coordinated activity of the nitrate transporters throughout the symplastic pathway during the nitrate transport along the root radius (**Figure 1A**). It also implicitly suggests a coordination in the bidirectionality of nitrate transporters functioning between epidermis membrane and vasculature tissues for nitrate xylem loading. The abrupt change in *v_in_* and *v_out_* after 0.5 mM external nitrate concentration led to high ^15^N translocation (*v_out_*) for the shoots growth. Since the external nitrate concentration impacts the shoots growth and capacitance for water (Orieux et al., 2019), this break in the translocation rate of ^15^N towards the shoots raises the question of whether a change in the sink-strength and/or N pools size of the shoots can explain this biphasic behavior.

### Early Removal of a Cotyledon Does Not Change the Biphasic Behavior of ^15^N Flux Patterns

To verify that the net ^15^N uptake (*v_in_*) depends on a coordinated regulation of nitrate transporters throughout the symplastic pathway but not on the sink-strength for N and/or N pools sizes of the growing cotyledons, the same experiment was carried out but we excised a single cotyledon 48 h after sowing before the autotrophic growth phase of the seedlings. To facilitate the comparison of data obtained between both experiments (exp1 and exp2), the overall trends of *v_in_*and *v_out_* (**Figure 4A**) of ^15^N flows after 120 h of treatment were normalized with regard to the velocity measured at 10 mM (**Figures 4B, C**). The results showed that the overall trends were strictly identical between both experiments. Again, the dynamic of *v_in_* and *v_out_* ^15^N flow profiles from the low to the high external nitrate concentrations was non-linear and the biphasic behavior of ^15^N translocation rate to the remaining cotyledon remained unchanged (**Figures 4B, C**). This indicates that this single cotyledon stored much more ^15^N than the two cotyledons of control plants. Despite an increase in its surface area, the remaining cotyledon could not completely compensate for the ablation-induced surface and volume loss compared to control plants (**Figure S3A**). Taken together, these results demonstrate that the ^15^N flux translocation rate is independent from the sink-strength and/or N pool size of the shoots. Moreover, the results underscore the existence of a strong coordination between the growth of the shoots’ surface area and roots’ length in response to the N flow rates. Indeed, if the coefficient corresponding to the relative compensatory increase in surface area of the remaining cotyledon is applied to half the root length of control seedlings, the estimated theoretical root length corresponds exactly to the root length of seedlings with an excised cotyledon (**Figure S3B**). These results also argue in favor of a control of the translocation rate by the roots instead of the shoots N demand since xylem unloading of nitrate into the shoots cells is not known as a control point.

### The Intersection of the *v_app_*and *v_out_* ^15^N Fluxes Reveals a Single Stationary State

Under nutritional steady-state conditions, the comparison of dynamic evolutions in *v_app_*and* v_out_* ^15^N fluxes makes it possible to graphically find stationary states. The velocity *v_app_* is the net flux of ^15^N stored in the roots corresponding to nitrate storage in vacuole and cytosol compartments and nitrate assimilation under N-reduced forms (**Figures 1A, B**). In fact, stationary states correspond to the intersection between both fluxes (see Mat and Meth) and are given for a specific external nitrate concentration noted $X^{*}={\left[NO_{3}^{-}\right]}_{ext}^{*}$ when:

d[N15]/dt=vapp−vout=0

**Figure 5:**
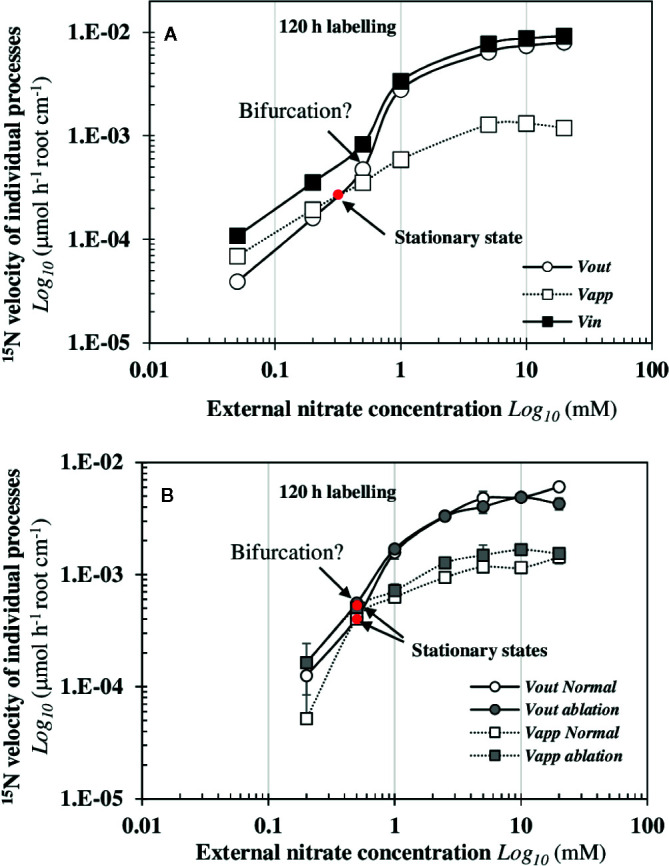
Graphically determination of a single stationary state induced by nitrate availability that precedes a dramatic bifurcation of the *v_out_* ^15^N flux towards the shoots. These graphs highlight the conditions of existence of a single stationary state and a bifurcation in two independent experiments after 120 h treatment with a NO3−15 labeling. The experiment 1 was performed with intact *B. napus* seedlings **(A)** and the experiment 2 **(B)** was carried out with intact seedlings (normal) and seedlings with one single cotyledon excised (ablation). Values are means ± SD of four repeats of four seedlings each.

Analysis of net *v_app_*and* v_out_* ^15^N flux rates after 120 h of ^15^N labeling showed that a single stationary state satisfies this condition for an external nitrate concentration around ${\left[NO_{3}^{-}\right]}_{ext}^{*}=0.3\;mM$ (**Figure 5A**). This value corresponds to an equilibrium between the net uptake rate of NO3−15 inside the root and net elimination rate of ^15^N to the shoots by its translocation (i.e., 50% of ^15^N allocation to the shoots). However, the analysis of ^15^N fluxes between 96 h and 120 h revealed another potential stationary state close to 0.5 mM (**Figure S2A**) that was probably masked due to an insufficient number of experimental data points between 0.2 and 1 mM nitrate (**Figure 5A**). This assumption was confirmed in the experiment with an excised cotyledon (**Figure 5B**). In normal seedlings (control) and in seedlings with a single excised cotyledon (ablation), the dynamic analysis of *v_app_*and* v_out_* ^15^N flux rates shows that the stationary state was reached for ${\left[NO_{3}^{-}\right]}_{ext}^{*}=0.5\;mM$. In both cases, the stationary state was strictly associated with the abrupt change in ^15^N translocation rate towards the shoots suggesting that this new stationary state was locally unstable (**Figure 5B**). However, in excised-cotyledon treatment, the early equilibrium in the ^15^N *v_in_*and *v_out_* flow rates between 0.2 and 0.5 mM of external nitrate concentrations (i.e., *v_ap_*_p_ and *v_out_* confounded) was not sufficient to increase the *v_out_* flux rate in favor of a dramatic translocation of ^15^N towards the shoots (i.e., a bifurcation). This result suggests again the possibility of two potential stationary states: one stable stationary state around 0.3 mM and another unstable stationary state around 0.5 mM (**Figure 5B**).

### Combined Allosteric Responses in ^15^N Fluxes Reveal That the Stationary State Is Locally Stable and Precedes a Dramatic Bifurcation in v_out_^15^N Fluxes

**Figure 6:**
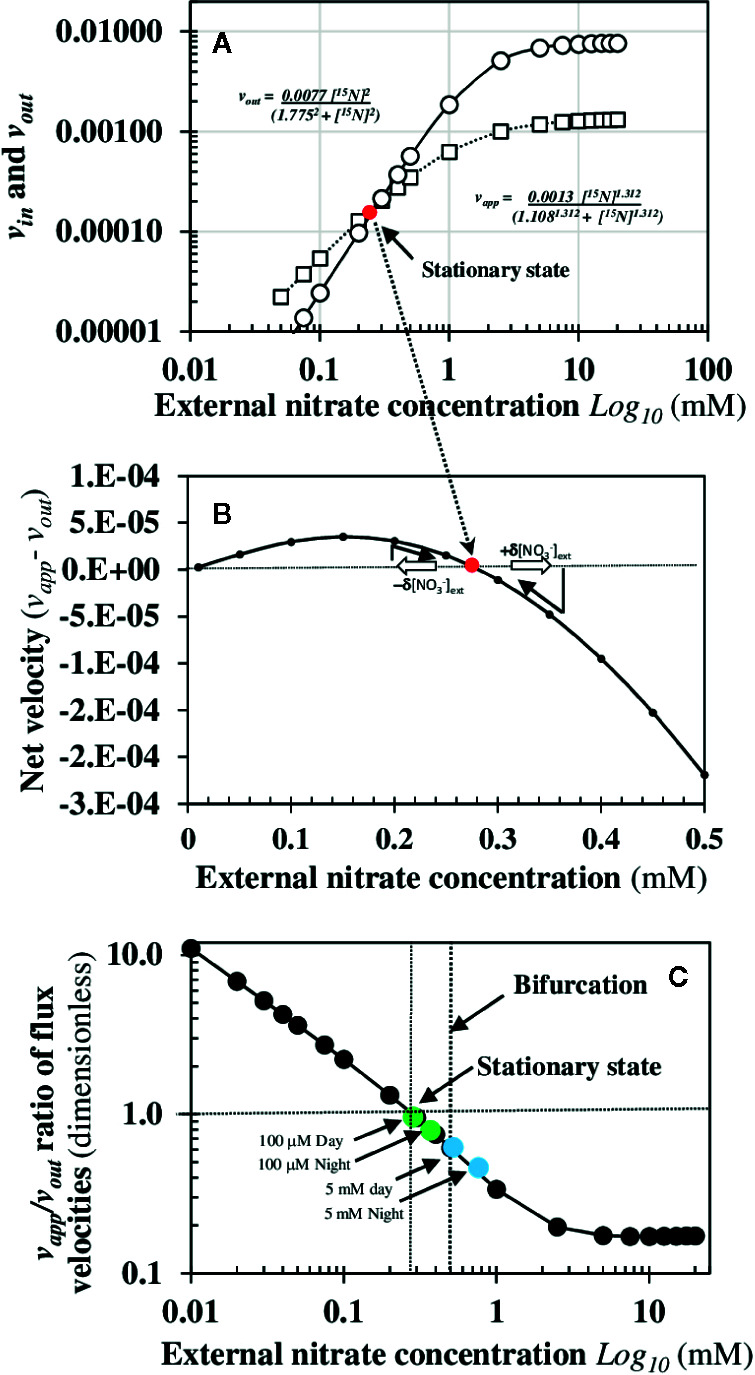
Determination of local stability of the single stationary state induced by nitrate availability. **(A)** Velocities of apparent $NO_{3}^{-}$ uptake rate (*v_app_*) and translocation rate (*v_out_*) of nitrate. The net ^15^N fluxes throughout the external nitrate concentrations were fitted with two empirical Hill equations indicated within the graph (r >0.99 and p<0.005). **(B)** Determination of the exact nitrate concentration ${\left[NO_{3}^{-}\right]}_{ext}^{*}=0.28\;mM$ for the stationary state when the net velocity of ^15^N flux estimated by *v_app_ – v_out_* function is equal to zero or d[^15^N]/dt = 0. **(C)** The velocities ratio *v_app_*/*v_out_* of the ^15^N fluxes reveal an increase in one order of magnitude in transporters activity from 0.28 (stationary state) to 0.01 mM of external nitrate concentrations compatible with the differential allosteric activities of distinct nitrate transporters such as *NRT2.1* and *NPF6.3/NRT1.1*. The green dots and blue dots refer to the *v_app_/v_out_* ratio obtained in an independent experiment where the rate of nitrate influx was measured at 100 mM and 5 mM during a day-night cycle (see **Table SI**).

To formalize the non-linear dynamics of *v_app_* and *v_out_* ^15^N fluxes in the roots, we used available molecular data on nitrate transporters and physiological studies of ^15^N and ^13^N fluxes. Nitrate is mainly taken up at the root epidermis cells by nitrate transporters such as *NPF6.3/NRT1.1* and *NRT2.1* that are induced by nitrate itself (Lejay et al., 1999). This positive loop of retroaction (called *ante* activation) acts at the transporter activity and gene expression level throughout the root symplastic pathway of nitrate transport to increase the carrier number within the cells plasma membrane and in turn the uptake of nitrate (Lejay et al., 1999). However, this auto-amplification loop is limited by the plasma membrane incorporation of transporters and the activity of H^+^-ATPases. Accordingly, to estimate *v_in_* and/or *v_out_*velocities*, *we assumed a sigmoidal dependence of the nitrate uptake or translocation rate with respect to exogenous and endogenous concentrations of nitrate. This type of relationship can be described by the empirical Hill’s equation according to:

$$v=V_{m}{\left[S_{j}\right]}^{nh}/({K_{0.5}}^{nh}+{\left[S_{j}\right]}^{nh})$$

where *nh* is the Hill’s number or cooperativity coefficient and defines the degree of cooperativity of the carrier (allosteric property). If *nh* is equal to 1, the process corresponds to Michaelis-Menten equation. *K_0.5_* is the concentration of the *S_j_* solute at half saturation when *v* = 0.5. The Hill’s coefficient is statistically interpreted as the number of molecules of ligand and/or substrate *S_j_* that fix simultaneously on an enzyme (Weiss, 1997). The estimation of *v_app_* velocity corresponding to the temporal concentration variation of the root endogenous ^15^N under nitrate or N-reduced forms (see *Materials and Methods*) is more complex because it is the sum of nitrate uptake and two elimination N fluxes: the sequestration rate of nitrate into the cytosol and/or vacuoles of root cells and the root assimilation rate of nitrate into N-reduced forms. These three types of reactions cannot be dissociated with this type of ^15^N labeling experiments (**Figure 1**). It is why estimated of the *v_app_* and *v_out_* ^15^N fluxes were made assuming the recycling of ^15^N remains constant between shoots and roots during the nitrate treatments. The sequestration and assimilation rates of nitrate are two processes regulated by the endogenous nitrate in the cytosol. Indeed, the sequestration rate depends on nitrate transporters functioning at the tonoplast membrane level that follow the Michaelis-Menten equation (Migocka et al., 2013), whereas nitrate assimilation rate depends at first on the nitrate reductase activity induced by nitrate. Therefore, we also assumed a sigmoidal dependence on *v_app_* flux with Hill’s equation since this function aggregates nitrate uptake, sequestration and assimilation fluxes. By the least square method, we were able to estimate the values of the different parameters (*V_m_, K_0.5_*, and *nh*) of the *v_app_* and *v_out_* equations with a r value superior to 0.99 with P<0.005 (**Figure 6A**). The dynamic study of ^15^N fluxes with this set of parameters allowed us to accurately estimate the external nitrate concentration corresponding to the single stationary state at $[NO_{3}^{-}]*=0.28\;mM$ when the v_app_ – v_out_ fluxes equal zero (**Figure 6B** and **Figures S3** and **S4**). Moreover, the velocities ratio *v_app_*/*v_out_* in ^15^N fluxes reveals an increase in one order of magnitude in transporter activity from 0.28 (stationary state) to 0.01 mM of external nitrate concentrations consistent with combined allosteric activities of distinct the nitrate transporters at low external nitrate concentration (**Figure 6C**). The stability of the stationary state was then studied by means of a mathematical algebraic approach of small perturbations in external nitrate concentrations around the stationary state $\left([NO_{3^{-}}\pm \delta [NO_{3^{-}}]\right)$ in order to test if it was locally stable or unstable (see appendix). The mathematical analysis demonstrated that this stationary state is locally stable, which means that a small increase or decrease in the external nitrate concentration brings the systems back to its stationary state. However, as previously stated, the dramatic ^15^N translocation to the shoots observed in ^15^N fluxes at 0.5 mM (**Figure 5**) suggests the presence of a bifurcation due either to another stationary state locally unstable or to the disappearance of the stable stationary state after higher variations in external nitrate concentrations. Because of the experimental limitation to separate endogenous NO3−15 and ^15^N-reduced forms by using the ^15^N tracer, a question remains whether the stationary state depends on endogenous or external nitrate concentrations.

### Abrupt Changes in Expression of BnNPF6.3/NRT1.1/CHL1 Nitrate Transporter Gene Were Closely Associated With the Bifurcation of ^15^N Translocation to the Shoots

**Figure 7:**
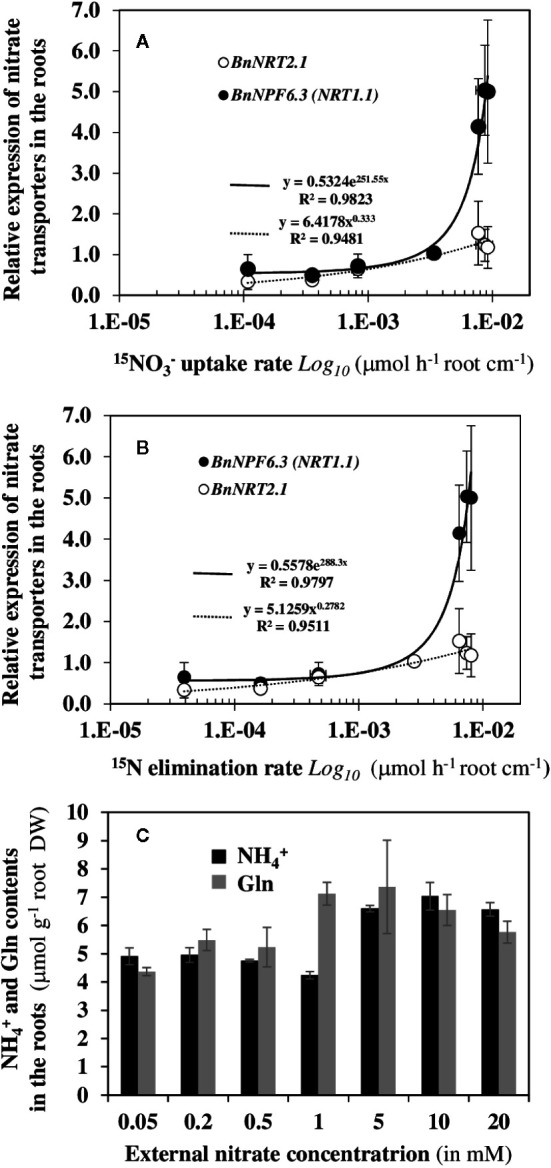
Expression changes in transcripts of nitrate transporters *BnNPF6.3/NRT1.1* and *BnNRT2.1* are correlated with dynamics of *v_in_* and *v_out_* ^15^N fluxes. The correlations were established between NO3−15 uptake rate *v_in_* **(A)** and ^15^N translocation rate *v_out_* **(B)** with expression levels of the nitrate transporter transcripts after 120 h treatment with a NO3−15 labeling. **(C)** Changes of free $\text{N}{\text{H}}_{4}^{+}$ and Gln contents in the roots of *B. napus* seedlings after 120 h of treatment with increasing external nitrate concentrations from 0.05 to 20 mM. Values are means ± SD of three repeats of four seedlings each.

Since nitrate NO3−15 uptake and translocation rates need nitrate transporters along the root symplastic pathway, we therefore examined whether the variations in ^15^N fluxes were linked to changes in gene expression of nitrate transporters after 120 h of NO3−15 labeling (**Figure 7**). Indeed, in *Arabidopsis AtNPF6.3*/*AtNRT1.1* and *AtNRT2.1* are both expressed in epidermis, cortex, endodermis and pericycle cell layers of the mature root (Guo et al., 2002; Nazoa et al., 2003; Girin et al., 2007). The results showed that changes in the ^15^N fluxes (*v_in_* and *v_out_*) were significantly correlated with the root transcriptional induction of the two nitrate transporter genes *BnNPF6.3/BnNRT1.1* and *BnNRT2.1* (**Figure 7**) indicating a transcriptional coordination of the two transporters along the symplastic pathway with the increase in ^15^N flow rates throughout changes in the external nitrate concentrations (**Figures 7A, B**). However, beyond 1 mM nitrate, the increase in expression of *BnNPF6.3/BnNRT1.1* was better correlated with changes in ^15^N flux translocation to the shoots (**Figure 7B**). The limited upregulation of *BnNRT2.1* when compared to *BnNPF6.3/BnNRT1.1* beyond 1 mM probably involved both the feedback repression by increase root contents of $\text{N}{\text{H}}_{4}^{+}$ and Glutamine (Gln) (**Figure 7C**) and the *BnNPF6.3/BnNRT1.1*-mediated repression by high nitrate demand as proposed by Krouk et al. (2006). These results indicate that *AtNPF6.3/AtNRT1.1* nitrate transporter is clearly involved in nitrate translocation rate (*v_out_*) to the shoots.

### The Translocation of NO3−15 and Water to the Shoots Requires the Concomitant Activity of the NRT2.1 and NPF6.3/NRT1.1 Transporters

**Figure 8:**
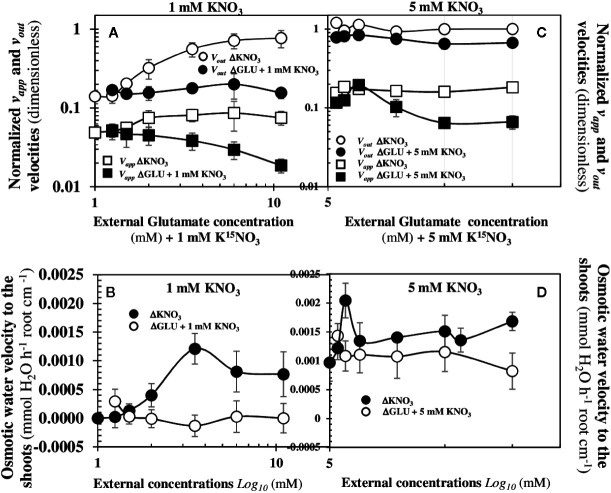
Changes in the Velocities of the *v_app_* and *v_out_* ^15^N Fluxes and the Osmotic Water Flux to the Shoots in Treated Seedlings with 1 and 5 mM KNO_3_ labeled with ^15^N. Effects of a co-treatment with 1 **(A)** or 5 mM **(C)** KNO_3_ with an increasing glutamate concentration (0.25; 0.5; 1; 2.5; 5; and 10 mM) on the *v_app_* and *v_out_* ^15^N fluxes measured by a labeling with ${\text{K}}^{15}\text{N}{\text{O}}_{3}$ (atom % ^15^N: 1%). Effects of a co-treatment with 1 **(B)** or 5 mM **(D)** KNO_3_ with an increasing glutamate concentration (0.25; 0.5; 1; 2.5; 5; and 10 mM) on the osmotic water flux towards the shoots for cotyledons growth Values are means ± SD of four repeats (= 4 Petri dishes) of four seedlings each.

To analyse whether the *NRT2.1* transporter is involved in the ^15^N *v_app_* and *v_out_* fluxes and the osmotic water flow to the shoots in response to nitrate availability, we re-examined another similar experiment (exp3) carried out under nutritional steady-state conditions from seedlings growing on agar plates (Leblanc et al., 2013). In this experiment, the activity and transcription of *NPF6.3/NRT1.1* and *NRT2.1* transporters were uncoupled by glutamate (Glu) treatments. Indeed, amino acids such as Glu specifically inhibit *NRT2.1* transporter activity and transcription levels but have no effect on the *NPF6.3/NRT1.1* transporter (Lejay et al., 1999; Loqué et al., 2003). Seedlings supplied with 1 mM or 5 mM ${\text{K}}^{15}\text{N}{\text{O}}_{3}$ were treated with increasing concentrations of Glu (0.25; 0.5; 1; 2.5; 5; and 10 mM). Indeed, as previously shown, beyond the 0.5 mM external nitrate concentration, the bifurcation of ^15^N fluxes (**Figure 4A**) and the structural reorganization at cellular and organ level (**Figure 3C**) are clearly engaged. The results showed the increase in Glu external concentrations under homogeneous supply of nitrate at 1 and 5 mM induced a collapse in the *v_app_* and *v_out_* ^15^N fluxes (**Figures 8A, C**). However, the associated-osmotic water flow for the shoots growth was completely abolished at 1 mM nitrate (**Figure 8B**) but not at 5 mM, where the bifurcation or ^15^N fluxes was still maintained but at lower levels (**Figure 8D**). These results demonstrate that the *NRT2.1* transporter is involved in *v_app_* and v_out_^15^N fluxes in low and high external nitrate concentrations and is mandatory for the nitrate priming effect that triggers the activity of *NPF6.3/NRT1.1* transporter for nitrate transport and translocation. Indeed, the glutamate-induced upregulation of *NPF6.3/NRT1.1* expression at 1 and 5 mM was unable to compensate for the glutamate-induced repression of the activity and expression of the *NRT2.1* transporter (see **Figure 5** in Leblanc et al., 2013). It also confirms the major role displayed by *NRT2.1* both in the increase in the root hydraulic conductivity (*L*p*_r_*) (Li et al., 2016) and osmotic water flux required for the shoot growth after nitrate supply (Leblanc et al., 2013; Le Ny et al., 2013; Orieux et al., 2019). Because the bifurcation in NO3−15 and water translocation rate to the shoots are both associated with the activity of nitrate transporters, these results raise the question of which fluctuating biological effectors or parameters are coordinated with the morphological break in apical-basal symmetry at the cellular and organ level.

### Changes in the Shoot Tryptophan Contents Follow a Power-Law Relationship Over a Wide Range of Nitrate External Concentrations

**Figure 9:**
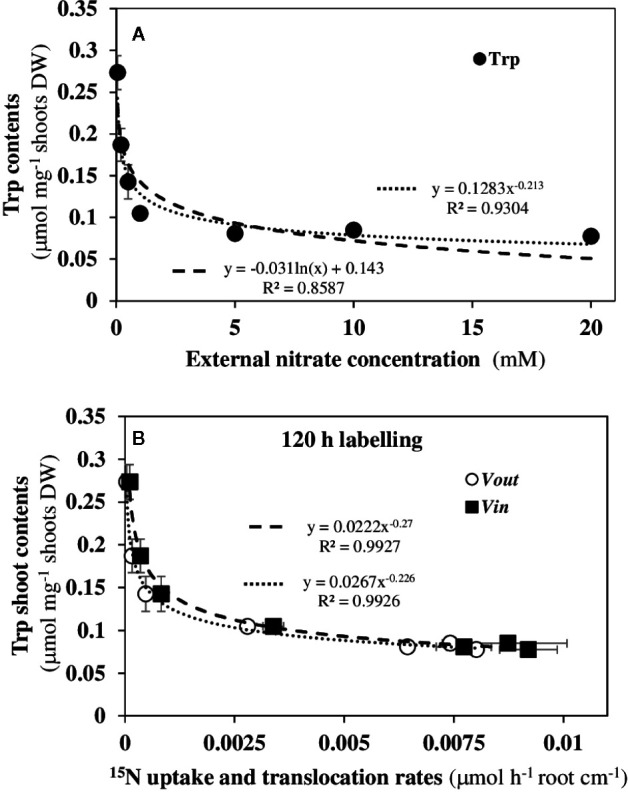
Power law relationships found between the contents of free tryptophan in the shoot tissues with external nitrate concentrations. **(A)** Power law *versus* logarithmic relationship in *B. napus* seedlings between the free Trp contents in the shoot tissues and nitrate availability under nutritional steady-state conditions. $\text{N}{\text{O}}_{3}^{-}$ concentration was the only changing variable and varied from 0.05 to 20 mM. **(B)** Power law relationships between free Trp contents in the shoots with ^15^N uptake rate (*v_in_*) and ^15^N elimination rate (*v_out_*). Values are means ± SD of three repeats of four seedlings each.

The auxin phytohormone (IAA) is a good candidate as a biological effector since in *Arabidopsis* IAA strongly induced *AtNPF6.3/NRT1.1* expression in vascular tissues of the roots and shoots in nascent organs (Guo et al., 2001 and Guo et al., 2002). Indeed, the location of *AtNPF6.3/NRT1.1* expression in roots and shoots is similar to the expression of auxin-reporter *DR5::GUS* probe in the presence or absence of nitrate (Guo et al., 2002). Furthermore, IAA is universally synthetized in plants by the indole-3-pyruvate (IPA) pathway from Trp *via* a conserved two-step reaction involving tryptophan aminotransferases (TAA/TAR) and YUCCA flavin monooxygenases (YUC). The expression patterns of TAA/TAR and YUC genes showed that they are commonly expressed in vascular tissues in shoots and roots of young *Arabidopsis* seedlings (Kasahara, 2016). Likewise, local IAA biosynthesis in the roots and transport from the shoots are essential to establish the root auxin gradient, to maintain the functioning of root meristem and to ensure the root plasticity to environmental cues (Brumos et al., 2018). Because Trp biosynthesis directly depends on nitrogen metabolism and is essential in IAA production *via* the IPA pathway, we recently examined the variations of Trp contents in shoot tissues in response to nitrate (Le Deunff et al., 2019a). When plotting shoot Trp contents against nitrate external concentrations on a *log-log* scale (**Figure S4**), a linear relationship is obtained which corresponds to a power-law relationship (*Y = kX^α^*) according to the following equation:

$${\left[Trp\right]}_{shoot}=k{\left[NO_{3}^{-}\right]}_{ext}^{\alpha }$$

where *α* is the law’s exponent and *k* is a constant (**Figures 9A, B**). This relationship was only found with Trp contents amongst all other free amino acids (AA) in the shoots (data not shown). According to this power law, 66.8% of the decrease in shoot Trp concentrations contents is caused by a 2.25% variation in external nitrate concentrations corresponding to 10-fold nitrate availability increase (from 0.05 to 0.5 mM). As shown in **Figure 3C**, this dramatic variation in Trp mainly affects the inversion in the root and shoot cell growth and occurs between 0.2 and 0.5 mM external nitrate. This cellular growth behavior stops at 1 mM external nitrate (i.e., 4.7% of nitrate increase and 86% decrease in Trp) and precedes the expansion by 2-fold of the cotyledon surface area between 1 and 20 mM external nitrate (see **Figure 2B**, Le Ny et al., 2013). As expected, the decrease in shoot Trp contents also exhibited a power-law relationship with the variation in cotyledon surface area demonstrating that Trp was mainly used to produce IAA necessary for the cotyledon cells and organ expansion (**Figure S5**). To establish a link with ^15^N fluxes, we then studied whether variations in IAA precursor contents were also correlated with *v_in_* and *v_out_* fluxes of ^15^N after 120 h of NO3−15 labeling (**Figure 9B**) or during a complete day-night cycle between 96 and 120 h (**Figure S6**). During the two labeling periods, statistically significant power-law relationships were found, suggesting that fluctuations in contents of Trp (IAA precursor) in the shoots was probably one of the main biological effectors involved in morphological changes associated with the ^15^N flux bifurcation.

### The Root/Shoot Ratio of Contents in Total Free Amino Acids Varies Also as the Power of Nitrate External Concentrations

**Figure 10:**
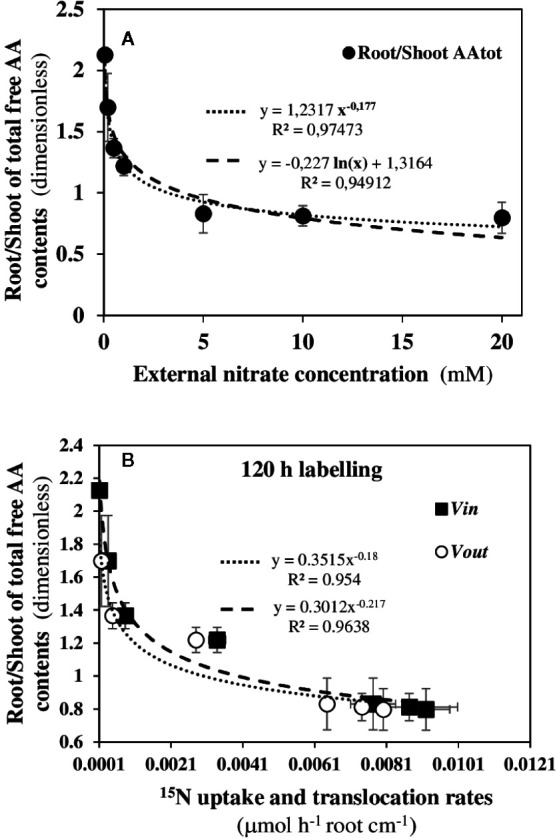
Power law relationships between root/shoot ratio of total amino acid contents and the dynamics of NO3−15 uptake rate (*v_in_*) and ^15^N translocation rate (*v_out_*) in response to nitrate availability. **(A)** Power law relationships obtained after 120 h of NO3−15 labeling between the root/shoot ratio of total AA contents with the external nitrate concentrations. **(B)** Power law relationships obtained after 120 h of NO3−15 labeling between the root/shoot ratio of total AA contents and the *v_in_* and *v_out_* ^15^N flux rates. *B. napus* seedlings were growing under nutritional steady-state conditions where $\text{N}{\text{O}}_{3}^{-}$ concentration was the only changing variable and varies from 0.05 to 20 mM. Values are means ± SD of three repeats of four seedlings each.

Although Trp is one of the least represented free amino acids in shoots of *Arabidopsis* (Watanabe et al., 2013), the biosynthesis pathway of its precursor, chorismate, is a major sink for C and N fluxes provided by N metabolism in relation to photosynthesis (Nune-Nesi et al., 2010). Therefore, we looked for possible coordination in the aminotransferase network involved in the primary N and C metabolism between the shoots and roots in response to nitrate. Because amino acids (AA) are the first products of nitrate reduction and assimilation and vary in both tissues depending on nitrate supply, we use the root/shoot ratio of total free amino acids as a synthetic parameter of global N status of the seedlings during nutritional steady-state in response to nitrate (**Figure 10**). Again, a statistically significant power-law relationship was found between this parameter and the increase in external nitrate concentrations (**Figure 10A**). The use of power-law relationship gave a better correlation than the exponential function previously used (Le Deunff et al., 2019a). Likewise, statistically significant relationships were also observed between the N status parameter (i.e., root/shoot ratio of total AA contents) and the *v_in_* and *v_out_* ^15^N fluxes (**Figure 10B**). These results indicate that the aminotransferase network involved in N and C metabolism at the whole plant level is highly coordinated and constrained between the shoots and roots in response to nitrate transporter activity induced by nitrate availability. Since Trp, IAA and free AA are molecules which circulate between the roots and shoots through the vascular system (i.e., xylem, phloem and stele parenchyma), relationships have also been sought between Trp contents and the root/shoot ratio of total AA contents (**Figure S7**). The statistically significant correlation found confirms that in steady-state nutritional conditions, Trp (i.e., IAA production) and tot AA biosynthesis are highly integrated at the primary N and C metabolism level in response to nitrate availability.

## Discussion

Unravelling the underlying mechanisms involved in the abrupt change in the uptake rate of $\text{N}{\text{O}}_{3}^{-}$, K^+^, $PO_{4}^{2-}$, $SO_{4}^{2-}$, and Cl^-^ ion transport in response to high fertilization levels is a fundamental issue for modern agriculture because this behavior is the basis for the success of the green revolution for increasing the yield potential of crop species. Here, we demonstrated by a dynamic non-linear analysis of ^15^N fluxes occurrence of a single stationary state, locally stable, at low external nitrate concentrations. This precedes a dramatic bifurcation in net ^15^N uptake and translocation rates to the shoots due to a marked crossed positive cooperativity in ^15^N fluxes. This may be due to the combined activity of nitrate transporters *BnNPF6.3/BnNRT1.1* and *BnNRT2.1.* Such outputs question the low- and high-affinity concept of nitrate transport deduced from the biphasic behavior of nitrate uptake isotherms based only on Michaelis-Menten equations. Ultimately, these findings have major ecological significance and offer new perspectives on nitrate uptake modeling.

### A Positive Cooperativity in Root Nitrate Transporters Activity Is Responsible for the Bifurcation in NO3−15 Fluxes

Mathematical analysis of non-linear dynamics of net ^15^N fluxes showed that the bifurcation in NO3−15 uptake rate (*v_in_*) and ^15^N translocation rate (*v_out_*) are under a positive cooperativity control of some specific nitrate transporters. Indeed, the Hill equation that best fit with the experimental data have a value of two for the Hill coefficient (*nh*). The only condition under which the Hill coefficient accurately estimates the number of binding sites is when a marked positive cooperativity is present, as in our experimental conditions (Weiss, 1997). This indicates the presence of two nitrate binding sites for the allosteric modification of transporters activity. Amongst the transporters mainly involved in root nitrate uptake, *NRT2* transporters are monomeric proteins whereas the *NPF6.3/NRT1.1* transporter is a monomeric protein able to dimerize after dephosphorylation of the threonine 101 (Ho et al., 2009). Moreover, it is clear from *NPF6.3/NRT1.1* crystallization studies that the dimerization is responsible for the increase in nitrate uptake capacity after conformational change (Sun et al., 2014; Parker and Newstead, 2014). A recent study demonstrated that intrinsic structural asymmetry between the protomers of the heterodimer *NPF6.3/NRT1.1* explains the allosteric functioning of the transporter by local conformational changes caused by nitrate binding on one or two protomers (Rashid et al., 2018). Therefore, our results confirm and demonstrate *in vivo* the involvement of the two nitrate binding sites required for the allosteric property of the *NPF6.3/NRT1.1* transporter that is consistent with a progressive increase in proportions of dimeric *versus* monomeric proteins in the plasma membrane and the bifurcation in transport and translocation capacities of *NPF6.3/NRT1.1* in response to high nitrate availability. This also explains the highly significant statistical correlation found between the *v_app_* and v_out_^15^N flux and the expression of *BnNPF6.3/BnNRT1.1* gene. To date, however, two other nitrate transporters, *NPF7.3*/*NRT1.5* and *NPF6.2/NRT1.4*, were shown to be involved in nitrate efflux to xylem (Lin et al., 2008; Léran et al., 2013). Indeed, studies with *Xenopus* oocytes have demonstrated that these three *NPF/NRT1* carriers behave like bidirectional transporters, but the ionic conditions that ensure the directional switch have not been established (Léran et al., 2013). This suggests that several transporters of the *NPF* family could be together involved in NO3−15 translocation, which is consistent with the sustained biphasic behavior of nitrate absorption of the mutants *nrt1.1* under high nitrate concentrations (see **Figure 7** in Liu and Tsay, 2003).

### How Does One Explain the Bifurcation of ^15^N Fluxes and the Osmotic Water Fluxes Required for Shoot Growth?

Since the root nitrate transport required transporters in every cell layer along the symplastic pathway and a preferred transfer of nitrate to the xylem, a major question to address is whether the change in the cooperativity in activity of the *NPF6.3/NRT1.1* transporter depends directly or indirectly on fluctuations of endogenous or exogenous concentrations of nitrate. Actually, physiological and molecular studies have clearly demonstrated with *nrt1* knockout mutants that the *NPF6.3/NRT1.1* transporter is either minimally or not involved in ^15^N or ^13^N nitrate uptake under low external nitrate concentrations (Glass and Kotur, 2013; Ye et al., 2019). By contrast, *NRT2* transporters such as *NRT2.1*, *NRT2.2, NRT2.4*, and *NRT2.5* are primarily involved (Lezhneva et al., 2014; Ye et al., 2019). In our study, these results are confirmed by the high expression of the *BnNRT2.1* gene at low nitrate concentrations and the velocities ratio *v_app_*/*v_out_* in ^15^N fluxes which reveals an increase in one order magnitude in transporters activity from 0.28 (stationary state) to 0.01 mM of external nitrate concentrations (**Figure 6C**). Moreover, a recent study of the functional properties of NPF6.3/NRT1.1 in xenopus ovocytes suggests that the nitrate influx and efflux by this transporter are also controlled by changes in endogenous nitrate concentrations (Noguero et al., 2018). Finally, application of external Glu to uncouple *NRT2.1* from *NPF6.3/NRT1.1* at the transcriptional and activity levels (exp3) indicated that the *NRT2.1* transporter is essential for nitrate translocation and osmotic water flux required for shoot growth in response to nitrate availability. Therefore, the modulation in *NPF6.3/NRT1.1* transporter activity could mainly depend on a priming effect exerted by the *NRT2* transporter in nitrate transport leading to the bifurcation in *v_in_* and *v_out_* nitrate fluxes induced by the *NPF6.3/NRT1.1* conformational change after dimerization. This hypothesis is consistent with both the combined cooperative properties of *NRT2* and *NPF6.3/NRT1.1* transporter activities and the involvement of *CIPK8* and *CIPK23* Ser/Thr kinases in the nitrate-induced transcriptional response of *NRT2.1* gene and the transport activity of *NPF6.3/NRT1.1* that are activated by Ca^2+^ sensors called CBL (Calcineurin B-like protein). Indeed, *CIPK8* leads to an upregulation on the expression of the gene *NRT2.1* in high nitrate concentrations range (> 1mM) whereas *CIPK23* provokes a downregulation of *NRT2.1* expression in low concentrations of nitrate (Hu et al., 2008; Ho et al., 2009). Therefore, the phosphorylation/dephosphorylation of *NPF6.3/NRT1.1* is probably controlled by the priming effect of *NRT2.1* on endogenous nitrate concentrations *via* Ca^2+^ signaling on the interactions of these two kinases modules.

### What Are the Regulatory Components Involved in Coordination Between the Nitrate Uptake at Root Epidermis and Nitrate Loading Into the Xylem?

The tight coordination between the nitrate uptake at root epidermis and nitrate xylem loading for nitrate translocation to the shoots is well illustrated by the strict parallelism in evolution in *v_in_* and *v_out_* ^15^N fluxes under nutritional steady-state conditions. However, it is easier to explain the nitrate uptake rate (*v_in_*) at root epidermis level with the combined cooperative properties of nitrate transporter activities rather than the translocation rate (*v_out_*). This raises the following question: what triggers the inversion of nitrate directional transport for the xylem loading at high external nitrate concentrations? A patch-clamp study with xylem-parenchyma protoplasts of barley roots identified a Quickly Activating Anion Conductance channel (X-QUAC) with the most important conductance for anions such as $NO_{3}^{-}$, Cl^-^, and malate. X-QUAC is activated in milliseconds and its activity is positively regulated by the decrease in the cytosolic concentration of Ca^2+^ (Köhler and Raschke, 2000; Köhler et al., 2002). In stellar cells of root corn X-QUAC-like activity has also been reported with similar properties, suggesting that X-QUAC ensures general function in xylem loading between plant species (Gilliham and Tester, 2005; Köhler and Raschke, 2007). Voltage dependence of X-QUAC possesses two gates, one opening at depolarization and the other with hyperpolarization that is modulated by the extracellular and not cytoplasmic $\text{N}{\text{O}}_{3}^{-}$. The increase in external nitrate concentrations induces a shift towards more hyperpolarized voltage allowing a positive feedback for the nitrate xylem loading. In fact, the general electric behavior of this channel corresponds exactly to a varistor also known as a surge arrester providing a mechanism to cope with a strong supply of nitrate to the roots (Thellier, 1973). This mechanism is consistent with abrupt and high increase in xylem nitrate concentrations (<35 mM) observed experimentally during the night and under low transpiring conditions (Herdel et al., 2001; Orieux et al., 2019) but also the presence of a potential binding site on the channel exposed to the apoplast. Although no link has been yet established between the X-QUAC and the *NPF/NRT* transporters such *NPF6.3/NRT1.1*, the allosteric dependence of nitrate on the *NPF6.3* transporter is intriguingly similar to the positive feedback regulation of X-QUAC exerted by the xylemic nitrate concentration (Köhler and Raschke, 2007; Rashid et al., 2018). Therefore, the combined cooperative properties of nitrate transporter activities at both ends of symplastic pathway could consistently work for both the absorption and translocation of nitrate. Furthermore, the auto-amplification loop (i.e., *ante* activation) in transporter activity for nitrate uptake and translocation is only limited by the enzymatic saturation around 20 mM. However, it does not exclude a possible retro-activation by specific effectors coming from the shoots compartment and transported *via* the phloem since the root vasculature tissues are the first to receive/perceive signal molecules such as ions, AA, sugars, peptides, hormones, and miRNA. In this respect, the presence of an H^+^-ATPase electrogenic pump in the plasmalemma of xylem-parenchyma cells supports a possible control of the loading and unloading of xylem anions through modulation of voltage differences across the membrane by regulatory factors such pH, AA and IAA (Köhler and Raschke, 2007). Nevertheless, the only known retro-activation belongs to the short-term nitrate uptake regulation (diurnal regulation) that is mainly explained by the positive effect of nitrate on photosynthesis regulation which in turn promotes N uptake (Delhon et al., 1995; Lejay et al., 1999; Girin et al., 2007; Lejay et al., 2008). However, the mechanisms and signaling cascades involved remain unclear, although several independent transcriptomic studies in *Arabidopsis* reported a high level of interaction between N, C, auxin, and cytokinin (CK) signaling pathways (for review see Ruffel et al., 2014). All together these results raise the fundamental question of whether the biphasic behavior observed in nitrate isotherms also corresponds to a thermodynamic bifurcation.

### Does the Biphasic Behavior of Nitrate Isotherms Also Correspond to a Thermodynamic Bifurcation?

It is clear from the combined cooperative properties of nitrate transporter activities throughout the root symplastic pathway under nutritional steady-state conditions that the high- and low-affinity concepts have strictly no scientific backgrounds. As previously demonstrated, when pure Michaelis-Menten enzymes are associated with a simple structure *in vitro* (e.g.,immobilization in a gel slade), they can produce different transport kinetics of Michaelis-Menten, sigmoidal and dual biphasic type. Therefore, in an integrated structure such as root, it is completely erroneous to conclude that the apparent kinetic parameters (*V_app_* and *K_app_*) are the actual molecular parameters of the enzyme-carriers (*V_m_* and *K_m_*). Indeed, a biphasic or sigmoidal kinetic curve can result from the functioning of a perfectly hyperbolic enzyme (see Vincent and Thellier, 1983 for the experimental and formal demonstration). Moreover, in essence, the complexity of the root catalytic device for absorbing nitrate along the symplastic path and its elimination by xylem loading masks the allosteric properties of the nitrate transporters and prevents any accurate experimental interpretation of nitrate isotherms. Indeed, the enzyme-substrate interpretation to explain the isotherm responses curves is based on several simplifying assumptions that do not hold such as: (i) nutritional conditions used are not far from the thermodynamic equilibrium and (ii) there is a constant export of the ion or substrate *S_j_* to the aerial parts over the duration of the experiment (see review, Le Deunff et al., 2016a). However, it is clear from accurate analyses of the literature that the first assumption is rarely met during most of the experimental conditions used (Britto and Kronzucker, 2001b; Le Deunff et al., 2016a). Recently, it was also demonstrated that conditions required for equilibrium conditions used with enzyme-substrate interpretation for ion uptake isotherm invalidate the Nernst-Planck equation (Dreyer and Michard, 2020). Likewise, it was previously demonstrated that ion transport, simple and complex enzyme reactions cannot be described by equilibrium models in biological systems (Ricard, 2006). The second assumption is also invalidated if we account for the translocation rate (*v_out_*) to the shoots of ^15^N or ^13^N tracers during isotherm building. This is well exemplified in a study were nitrate influx measurements for 5 min at 100 μM and 5 mM were performed throughout the day-night cycle at 3 h intervals after a pre-acclimation for 1.5 h with 100 μM and 5 mM unlabeled nitrate (**Table SI**). The results revealed that the ^15^N translocation rate at 5 mM was about twice as high as 100 μM, regardless of the period of light or darkness (**Table SI**). Moreover, the ratio of *v_app_*/*v_out_* for influx measurements at 100 μM and 5 mM demonstrates that the bifurcation is close to occur (see green dots in **Figure 6C**) at 100 μM nitrate measurements and had occurred at 5 mM nitrate (see blue dots in **Figure 6C**). Therefore, re-examination of non-linear dynamics of ^15^N or ^13^N fluxes (*v_app_* and *v_out_*) is urgently needed when building nitrate isotherms, and special attention must be paid to the nutritional conditions used and *v_app_* and *v_out_* fluxes in order to unravel their biphasic behavior.

### How Can Root and Shoot Morphological Changes at the Cellular Level Be So Closely Associated With the Bifurcation of ^15^N Fluxes?

The power-law relationship found between variations in cotyledon surface area and decline in shoot Trp contents is consistent with IAA production through the IPA pathway for the shoot cells expansion and increase in the cotyledons’ surface area. However, biosynthesis of IAA alone cannot induce cell expansion without K^+^ uptake (Claussen et al., 1997; Philippar et al.,1999; Christian et al., 2006; Osakabe et al., 2013). Furthermore, bifurcation in *v_out_* ^15^N flux is also consistent with a simultaneous xylem loading of K^+^ to balance the negative charge of nitrate. Indeed, the electroneutrality of xylem salt export is ensured by the coupling between K^+^ and $\text{N}{\text{O}}_{3}^{-}$ fluxes (Herdel et al., 2001). Additionally, regulation of $\text{N}{\text{O}}_{3}^{-}$ and K^+^ transporters are under the same regulatory CBL-CIPK complex (Calcineurin B-like-Interacting Protein Kinase) in response to Ca^2+^ signaling and $\text{N}{\text{O}}_{3}^{-}$ and K^+^ concentration levels (Ho et al., 2009; Li et al., 2017; Coskun et al., 2017; Raddatz et al., 2020). In this respect, recent molecular studies with double mutants of NPF6.3/*NRT1.1* (*nrt1.1*) and K^+^ transporters (*akt1, hak5-3, kup7, and skor2*) have demonstrated functional interactions between these transporters in both the epidermis and central vasculature that coordinate and balance the distribution of these ions between roots and shoots organs (Fang et al., 2019). Taken together these results reinforce the assumption of a tight relation between cellular morphological bifurcation in the roots and shoots and ^15^N fluxes depends on a concomitant translocation of K^+^ and $\text{N}{\text{O}}_{3}^{-}$. The afflux of K^+^ in the shoots should act with IAA to induce the shoot cell expansion. The obvious follow-on question is: what triggers the IAA biosynthesis *via* IPA pathway in shoot tissues?

Short-term changes in CK biosynthesis, signaling and status in response to nitrate support the assumption that this hormone mediated rapid changes in cell division and expansion associated with nitrate-induced root and shoot morphological inversion (Takei et al., 2002; Kiba et al., 2010). Indeed, transcriptomic studies have well demonstrated that the IPT3 (IsoPentenyTransferase 3) gene involved in CK biosynthesis is strongly and rapidly induced by nitrate treatments (Scheible et al., 2004; Wang et al., 2004). In *Arabidopsis*, CK status regulates tryptophan aminotransferase 1 (TAA1) gene expression of IPA pathway *via* type B ARRs (Response Regulators) transcription factors: ARR1, ARR10, and ARR12 that bind to two different sequence elements in the TAA1 promoter (Zhou et al., 2011; Yan et al., 2017). Finally, osmotic turgor necessary for the shoot cell expansion needs a high osmotic water flow. As previously demonstrated, the increase in external nitrate concentrations significantly enhanced the root water uptake and shoots capacitance irrespective of the transpiration rate (Le Ny et al., 2013; Orieux et al., 2019). Since the rate-limiting step of water uptake is the mechanical resistance of the cell wall in growing tissues (Boyer, 1985), the high translocation of nitrate and potassium should result in lower osmotic potential and, in turn, higher turgor which should trigger growth of the shoot cells in response to increased IAA concentrations (Le Ny et al., 2013).

### Ecological Significance of N Uptake Rate Bifurcation in Response to Nitrate

The low concentration of nitrate on the vicinity of roots required to reach a stable stationary state allows plants to balance the N allocation between the roots and shoots as well as ensure a basic flow of N for shoot development in non-anthropized low N soils. However, the small increase in nitrate concentration required to bypass the stable stationary state and to trigger bifurcation is probably the major mechanism of nitrate sensitivity for plants to optimize their N uptake efficiency (NUptE) in order to complete their life cycle (e.g., g of seeds/g of N). Consistently, this thermodynamic mechanism allows plants to abruptly react and make the best use of a fertilization treatment or a nitrate rich patch caused by mineralization to increase locally the nitrate uptake capacity and in turn the shoot development. Even under unfavorable conditions or in absence of N fertilization, plants will maximize the rate of N translocation to the aerial part in response to small fluctuations of soil nitrate concentrations around the stationary point. At the same time, this mechanism limits the osmotic water flow towards the shoots for the benefit of elongation and development of lateral roots involved in soil foraging. Furthermore, the positive amplification loop (i.e., *ante*-activation) of such a mechanism suggests that the system first adjusts to nitrate supply rather than the shoot N demand. With such a mechanism, the saturation of nitrate uptake is reached for high concentrations of nitrate (> 20 mM) when other ions such as K^+^ and ${\text{PO}}_{4}^{2-}$ are not limiting. This is also in agreement with a recent transcriptomic study on *Arabidopsis* seedlings growing in hydroponic conditions under 0, 1, 10, and 60 mM external nitrate where the transcription rates of nitrate-induced genes were nitrate dose-dependent under the control of a cascade of specific transcription factors (Swift et al., 2020). Taken together, these results explain why during high nitrate fertilization after bifurcation of N flux to the shoots, the growth and production performances of plant aerial parts is often associated with luxury consumption of nitrogen. The combined cooperative mechanism between nitrate transporter activities is also consistent with the kinetics of N uptake of *B. napus* plants under field conditions during a whole growth cycle where the increase in fertilization level induces an over-accumulation of N. Indeed, kinetic analysis of the temporal trajectory of nitrate uptake rate for three levels of nitrate fertilization (N0 = 0 kg N ha^-1^; N1 = 135 kg N ha^-1^; and N2 = 235 kg N ha^-1^) showed that N accumulation was mainly due to adjustment in the N uptake capacities instead of root proliferation (Malagoli and Le Deunff, 2014; Le Deunff et al., 2019b). Because under field conditions, plants rarely reach N satiety, the results also indicate that this mechanism associated with the retro-activation of nitrate uptake by C products provided by photosynthesis generally exceeds mechanisms of retro-inhibition by amino acids before the foliar senescence. Therefore, it might be interesting to use non-linear dynamic analysis of ^15^N fluxes on seedlings growing under nutritional steady-state conditions to compare different genotypes of crop species possessing different NUptE in order to establish their stationary state and bifurcation pattern. From a fundamental point of view, the use of altered *Arabidopsis* mutants in nitrate reduction (e.g., *nia1* and *nia2*), nitrate vacuole sequestration (e.g., *clca* and *vha-a2*) and translocation (e.g., *nrt1.1*, *ntr1.5*, *nrt1.4*, and *nrt1.8*) would allow us to decipher other major players involved in the stationary state and flux bifurcation (Scheible et al., 1997; Hu et al., 2008; Lin et al., 2008; Han et al., 2016). Indeed, double null mutants of nitrate reductase (*nia1nia2*) in tobacco showed a very strong accumulation of nitrate towards the shoots from 0.2 mM external nitrate concentration compared to the control plants suggesting that the regulation of root assimilation rate of nitrate is highly coordinated with the nitrate translocation rate (Scheible et al., 1997; Britto and Kronzucker, 2003). Likewise, in *B. napus* seedlings with high NUptE, the activity of V-ATPase and V-PPase protons-pumps involved in nitrate vacuole sequestration was reduced whereas nitrate concentration in the xylem sap was increased (Han et al., 2015 and Han et al., 2016).

### Nitrate Uptake Modeling Perspectives

Because the nitrate influx measurements are most of the time carried out under conditions far from thermodynamic equilibrium, it would be better to establish the isotherms in nutritional steady-state conditions. Indeed, the cross cooperative properties in activity of nitrate transporters along the root symplastic pathway seriously challenge scientific grounds for the concept of high and low affinity for nitrate uptake (see also Le Deunff et al., 2016a; Dreyer and Michard, 2020). Therefore, the hyperbola equations used so far to fit experimental data to build the isotherms could be judiciously replaced by a single sigmoidal equation to model the *v_in_* flux in the N uptake models. Additionally, the cross-combination of such isotherms with the effect of photosynthetically active radiation (PAR), temperature, photoperiod and relative humidity should refine the current N uptake models (Le Deunff and Malagoli, 2014b; Malagoli and Le Deunff, 2014). In addition, the building of isotherms throughout a day-night cycle under steady-state nutritional conditions should also be seriously considered. Indeed, the nitrate uptake over 24 h aggregates all the pleotropic signals involved in the regulation of N uptake, such as sugars provided by photosynthesis, and allows an up-scaling of hour to day in N-uptake models (Malagoli and Le Deunff, 2014).

## Conclusion

The complexity of the data provided by transcriptomic and system biology studies are unable to build a clear holistic view and a simple mechanistic interpretation of the event sequence involved in responses to nitrate availability (Vidal et al., 2020). Analysis of non-linear dynamics of ^15^N fluxes offers an alternative and integrated approach to understand the major underlying processes. In simple terms, this corresponds to the difference between a road map and the car fluxes caused by traffic. Here, the analysis revealed the presence of a locally stable stationary state that precedes a dramatic bifurcation in ^15^N uptake and translocation rates. Moreover, the results suggest that K^+^ and water translocation associated with IAA biosynthesis in shoots probably plays a major role in nitrate-induced apical-basal break in symmetry between the root and shoot cell expansion. Therefore, more accurate analyses of non-linear dynamic ^15^N fluxes coupled to variations in external concentrations of K^+^ tracer should be done in order to determine if multi-stationary states and/or specific changes could occur with two changing variables (i.e., $\text{N}{\text{O}}_{3}^{-}$ and K^+^) as well as hysteresis phenomenon (i.e., the property of a system whose evolution does not follow the same path depending on whether an external cause (here $\text{N}{\text{O}}_{3}^{-}$ and/or K^+^) increases or decreases). Likewise, accurate day-night cycle analyses of dynamics of ^15^N and water fluxes (Orieux et al., 2019) associated with the use of specific mutants should corroborate the importance of some transporters in nitrate uptake, sequestration and translocation under different water flow regimes.

## Data Availability Statement

All datasets presented in this study are included in the article/**Supplementary Material**.

## Author Contributions

ED conceived the article and wrote the first draft. ED, PB, and CD designed research and performed the experiments. ED performed ^15^N analyses, CD performed UPLC analyses, and PB performed Licor 6 400 analyses. ED, PM, and JL analyzed data. ED, JL, CD, and PM revised the final version and approved for submission.

## Conflict of Interest

The authors declare that the research was conducted in the absence of any commercial or financial relationships that could be construed as a potential conflict of interest.

## Appendix. Mathematical Determination of the Stability of the Stationary State

The dynamic behavior of a system with a single changing independent variable *X* (here $X={\left[NO_{3}^{-}\right]}_{ext}$), is given by a specific integral solution from the non-linear differential equation:

$$X’(t)=dX/dt=f(X,(p_{k}))$$

where *p_k_* are the parameters of the system. The specific values that satisfy:

$$dX/dt=f(X,\;(p_{k}))=0\;or\;f(X)=0$$

are named $X^{*}={\left[NO_{3}^{-}\right]}_{ext}^{*}$ and correspond to stationary states which are the roots of the non-linear equation. In our case:

$$f(X^{*})=d{[}^{15}N/dt=v_{app}-v_{out}=0$$

To extract the roots *X^*^* of this new function *f(X)* it is necessary to apply numerical or algebraic methods. Since the system is in stationary state *X^*^*, if we apply a small perturbation *ΔX_0_*, the system is now in the new state $X(0)=X^{*}+\Delta X_{0}$. To determine the temporal evolution of the system after this small perturbation, it is possible to linearize the non-linear equation *f(X)* from Taylor’s series development limited to the first order (for details see Laurent, 2013). This conduct to the non-linear differential equation:

$$d(\Delta X)/dt={\left(df/dX\right)}^{*}\Delta X$$

with its particular integral solution given by:

$$\Delta X(t)=\Delta X_{0}e^{(df/dX)*t}$$

According to this solution, the stationary state *X^*^ = [NO_3_^-^]^*^* will be stable if *(*d*f/*d*X)^*^* < 0, in this case the system returns to *X^*^.* The stationary state $X^{*}={\left[NO_{3}^{-}\right]}^{*}$ will be unstable if *(*d*f*/d*X)^*^* > 0, in this case the system deviates from *X^*^*.

Because *f(X)* is a velocity function, *f’(X)* is in fact an acceleration corresponding to a second derivative. In our analysis, the *v_app_* and *v_out_* ^15^N flux velocities were fitted with Hill functions according to:

$$f(x)=v=V_{m}{\left[S_{j}\right]}^{n}/(K_{0.5}^{n}+{\left[S_{j}\right]}^{n})$$

with the apparent uptake rate (*v_app_*) and translocation rate (*v_out_*) which are given respectively by:

vapp=Vm[Sj]n/(K0.5n+[Sj]n)=0.0013[N15]1.312/(1.1081.312+[N15]1.312)

vout=Vm[Sj]n/(K0.5n+[Sj]n=0.0077[N15]2/(1.7752+[N15]2)

Where *Vm, K_0.5_* and *n* are parameters and *S_j_* is changing variable (here, ${\left[NO_{3}^{-}\right]}_{ext}$ *)*. We can then estimate the first derivative of each of Hill equation used to fit the *v_app_* and voutN15 flux velocities according to:

$$f(x)=U/V=>f’(x)=U’V-UV’/V^{2}$$

with

$$U=V_{m}{\left[S_{j}\right]}^{nh}=a{\left[X\right]}^{n}$$

$$V=K_{0.5}^{nh}+{\left[S_{j}\right]}^{nh}=b^{n}+{\left[X\right]}^{n}$$

where the parameters *V_m_*, *K_0.5_* and *n* were replaced by *a*, *b*, and *n *and *S_j_* by *x*, respectively.

$$U’=na{\left[X\right]}^{n-1}\;and\;V’=n{\left[X\right]}^{n-1}$$

$$\begin{array}{l} =>\;U’V–UV’\;=\;na{\left[X\right]}^{n–1}\left(b^{n}+\;{\left[X\right]}^{n}\right)\;-\;a{\left[X\right]}^{n}\left(n{\left[X\right]}^{n–1}\right)\\ =nab^{n}{\left[X\right]}^{n–1}+\;na{\left[X\right]}^{2n–1}-na{\left[X\right]}^{2n–1}=\;nab^{n}{\left[X\right]}^{n–1}\\ \;=>V^{2}=\;{\left(b^{n}+\;{\left[X\right]}^{n}\right)}^{2} \end{array}$$

From the derivatives of the two Hill functions and by replacing the constant values *a, b, n_1_* and *c, d, n_2_*, obtained from the least square method of ^15^N flux of experimental values (excel solver), it is possible to calculate the sign of *f(x)* derivative for *f(x)* = *v_app_- v_out_ = 0* according to:

f(x) =U–V=>f’(x) =U’−V’

that gives:

$$f’\left(x\right)\;=\;n1ab^{n1}{\left[X\right]}^{n1–1}/{\left(b^{n1}+\;{\left[X\right]}^{n1}\right)}^{2}–\;n2cd^{n2}{\left[X\right]}^{n2–1}/{\left(d^{n2}+\;{\left[X\right]}^{n2}\right)}^{2}$$

that can be rearranged in

$$\begin{array}{c} f’\left(x\right)\;=(\;n1ab^{n1}{\left[X\right]}^{n1–1}.{\left(d^{n2}+\;{\left[X\right]}^{n2}\right)}^{2}–\;n2cd^{n2}{\left[X\right]}^{n2–1}.(b^{n1}\\ {+\;{\left[X\right]}^{n1})}^{2})/{\left(b^{n1}+\;{\left[X\right]}^{n1}\right)}^{2}.{\left(d^{n2}+\;{\left[X\right]}^{n2}\right)}^{2} \end{array}$$

As the denominator of this function is always positive, the positive or negative value will depend on *f’(x)* numerator term for *x^*^.* Numerical calculations with the values of the constants *a, b, n_1_* and *c, d, n_2_*show that this term is always negative for $x^{*}={\left[NO_{3}^{-}\right]}^{*}=0.28\;mM$, demonstrating that the stationary state is locally stable but that its disappearance for high *ΔX_0_*perturbations**precedes a bifurcation.
