# Supplementary material for: Combined Allosteric Responses Explain the Bifurcation in Non-Linear Dynamics of 15N Root Fluxes Under Nutritional Steady-State Conditions for Nitrate
Source: Front Plant Sci. 2020 Aug 28;11:1253. doi: 10.3389/fpls.2020.01253 (PMC7770280; doi:10.3389/fpls.2020.01253)
Supplement: Supplementary file 1 [file Presentation_1.pptx]

## Slide 1
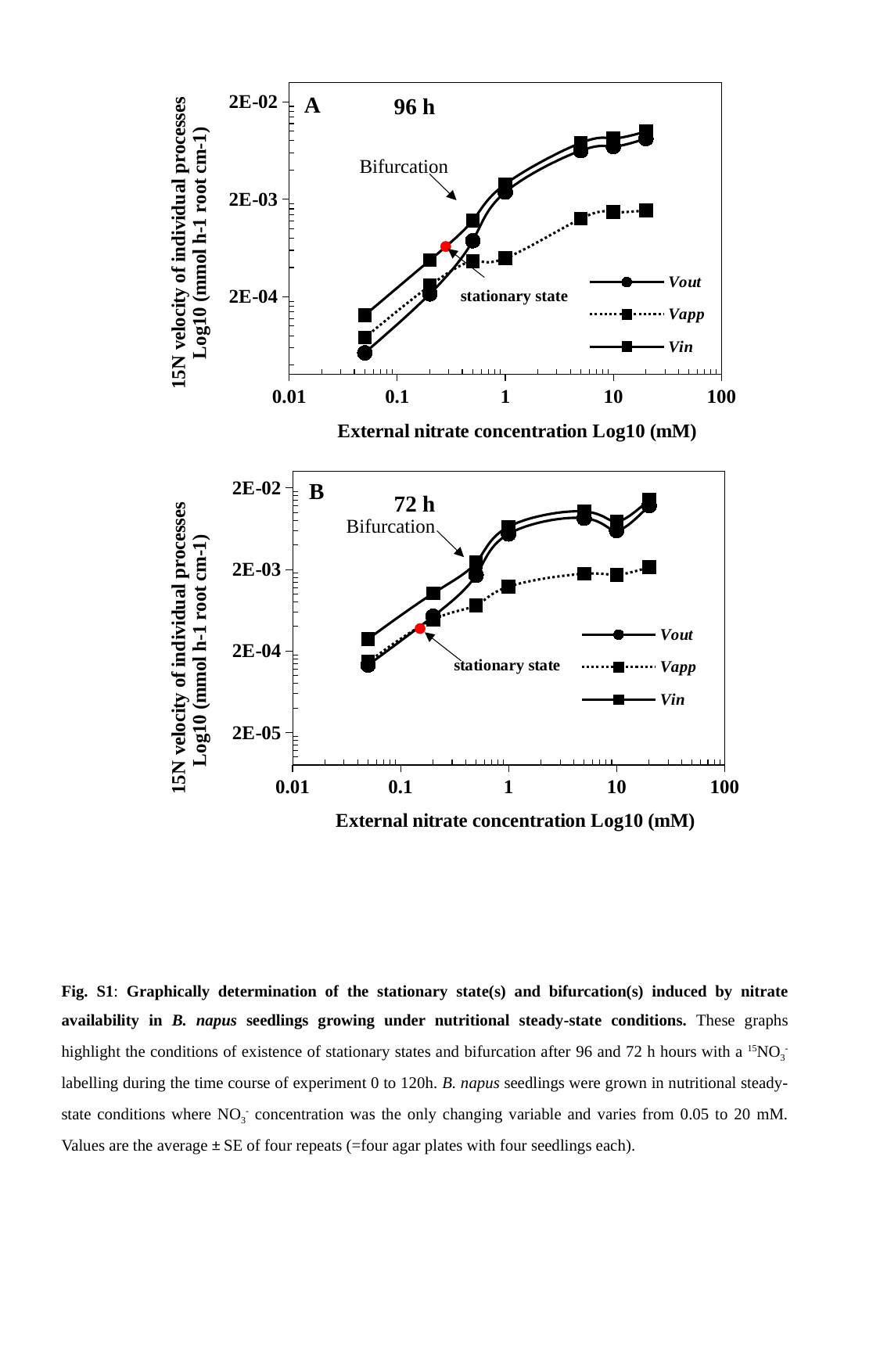

### Chart
| Category | | | |
|---|---|---|---|A
96 h
Bifurcation
stationary state
### Chart
| Category | | | |
|---|---|---|---|B
72 h
Bifurcation
Fig. S1: Graphically determination of the stationary state(s) and bifurcation(s) induced by nitrate availability in B. napus seedlings growing under nutritional steady-state conditions. These graphs highlight the conditions of existence of stationary states and bifurcation after 96 and 72 h hours with a 15NO3- labelling during the time course of experiment 0 to 120h. B. napus seedlings were grown in nutritional steady-state conditions where NO3- concentration was the only changing variable and varies from 0.05 to 20 mM. Values are the average ± SE of four repeats (=four agar plates with four seedlings each).

## Slide 2
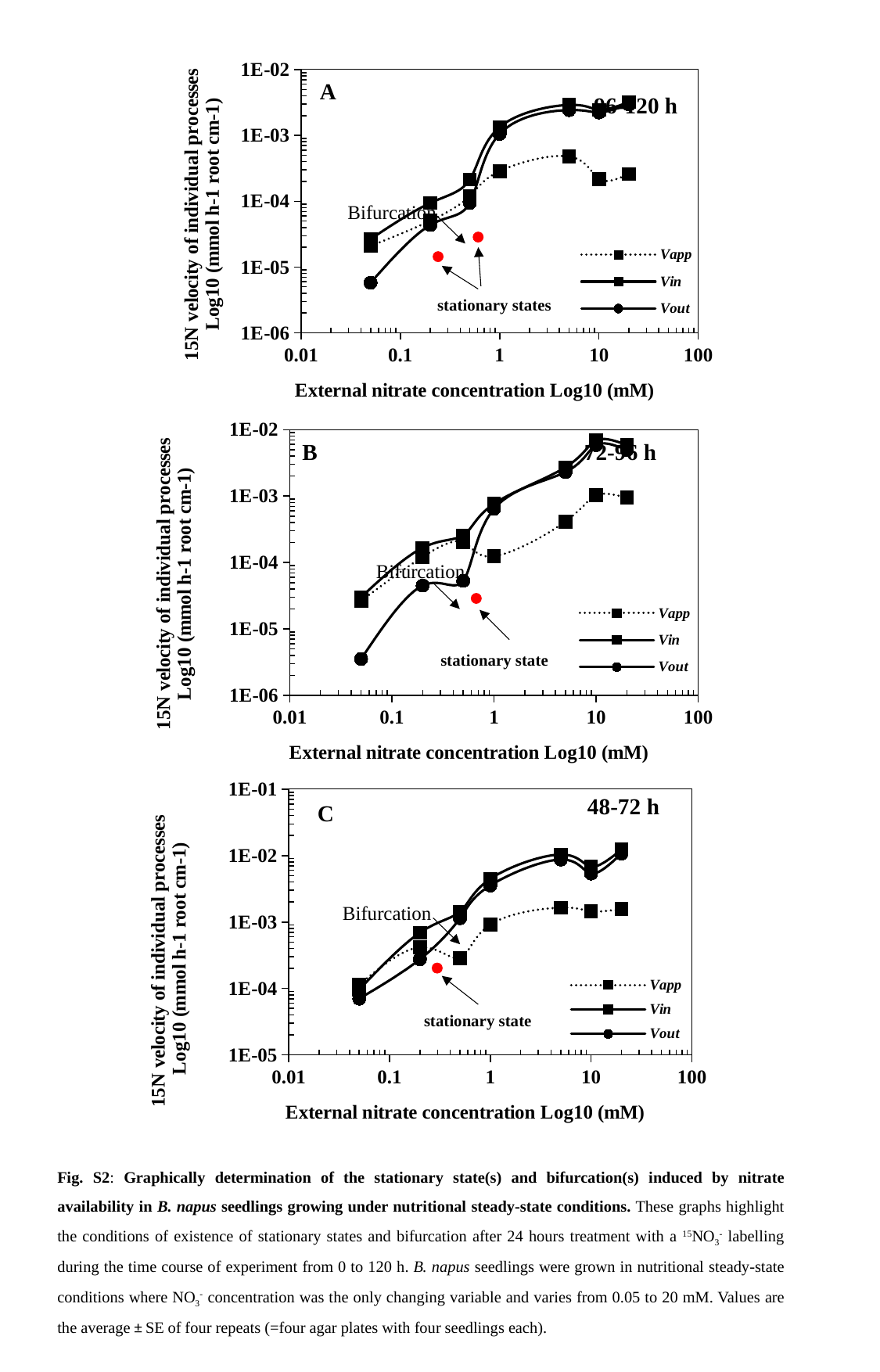

### Chart
| Category | | | |
|---|---|---|---|A
96-120 h
Bifurcation
stationary states
### Chart
| Category | | | |
|---|---|---|---|72-96 h
B
Bifurcation
stationary state
### Chart
| Category | | | |
|---|---|---|---|48-72 h
C
Bifurcation
stationary state
Fig. S2: Graphically determination of the stationary state(s) and bifurcation(s) induced by nitrate availability in B. napus seedlings growing under nutritional steady-state conditions. These graphs highlight the conditions of existence of stationary states and bifurcation after 24 hours treatment with a 15NO3- labelling during the time course of experiment from 0 to 120 h. B. napus seedlings were grown in nutritional steady-state conditions where NO3- concentration was the only changing variable and varies from 0.05 to 20 mM. Values are the average ± SE of four repeats (=four agar plates with four seedlings each).

## Slide 3
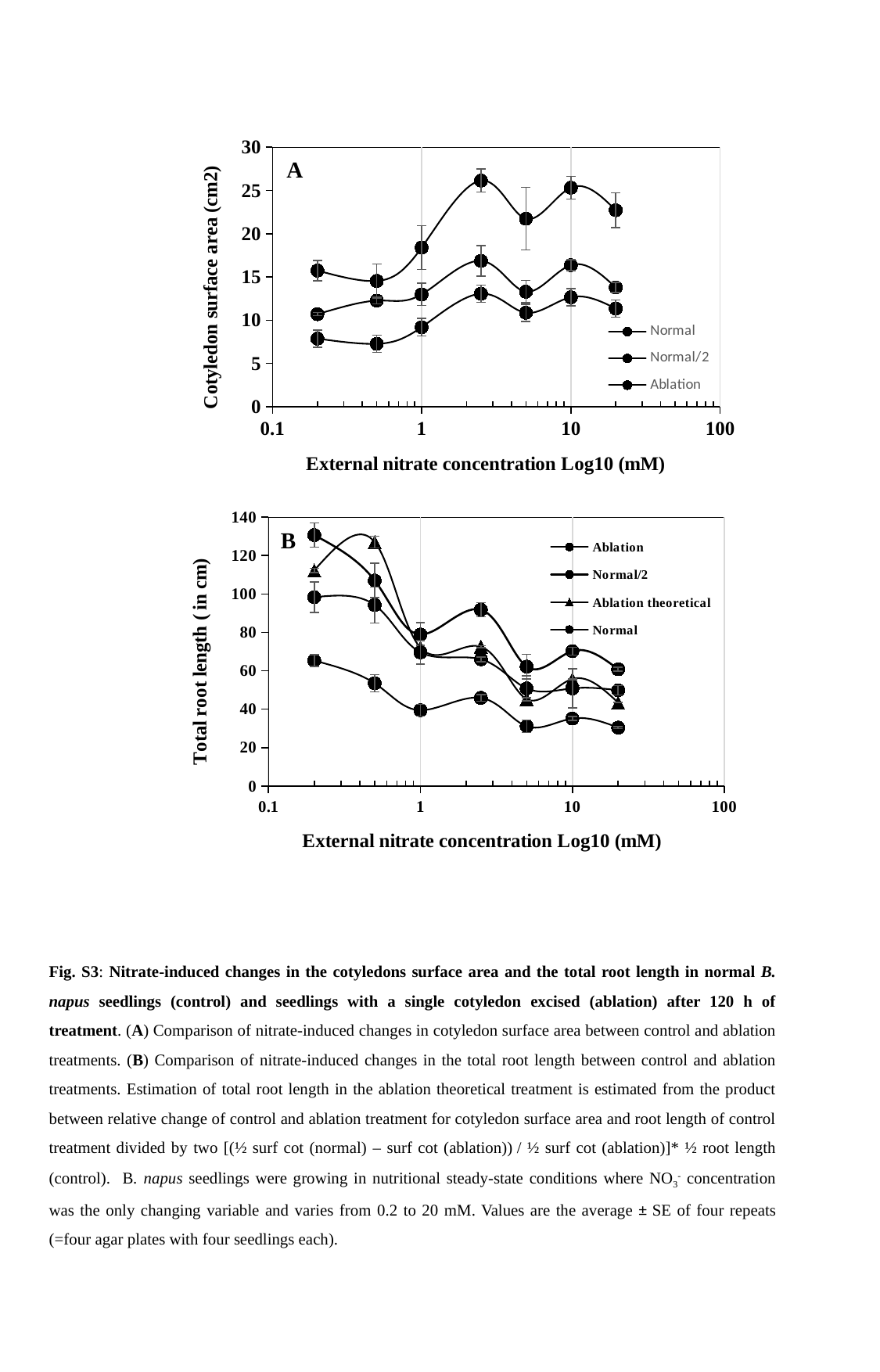

### Chart
| Category | | | |
|---|---|---|---|A
### Chart
| Category | | | | |
|---|---|---|---|---|B
Fig. S3: Nitrate-induced changes in the cotyledons surface area and the total root length in normal B. napus seedlings (control) and seedlings with a single cotyledon excised (ablation) after 120 h of treatment. (A) Comparison of nitrate-induced changes in cotyledon surface area between control and ablation treatments. (B) Comparison of nitrate-induced changes in the total root length between control and ablation treatments. Estimation of total root length in the ablation theoretical treatment is estimated from the product between relative change of control and ablation treatment for cotyledon surface area and root length of control treatment divided by two [(½ surf cot (normal) – surf cot (ablation)) / ½ surf cot (ablation)]* ½ root length (control). B. napus seedlings were growing in nutritional steady-state conditions where NO3- concentration was the only changing variable and varies from 0.2 to 20 mM. Values are the average ± SE of four repeats (=four agar plates with four seedlings each).

## Slide 4
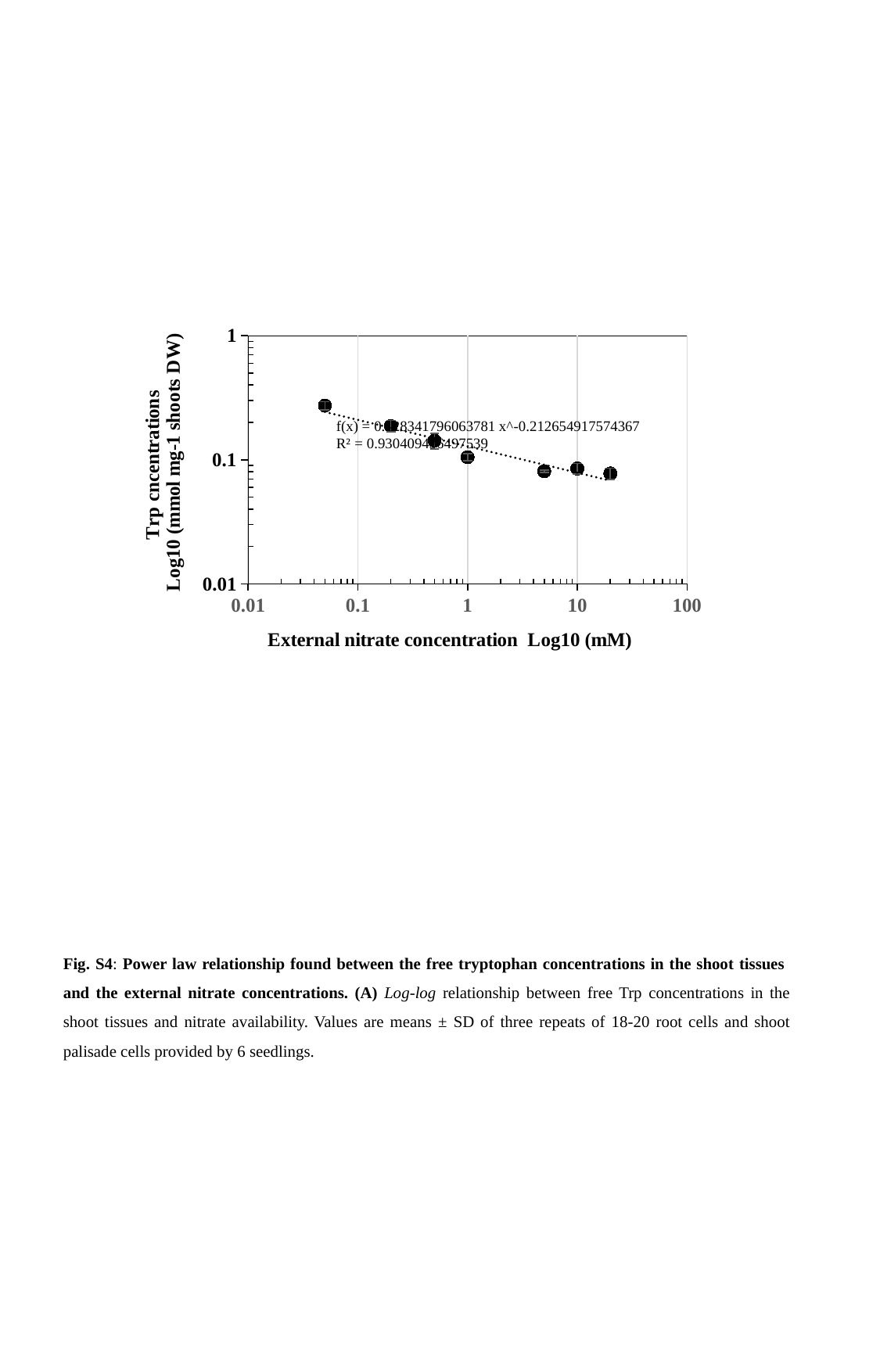

### Chart
| Category | |
|---|---|Fig. S4: Power law relationship found between the free tryptophan concentrations in the shoot tissues and the external nitrate concentrations. (A) Log-log relationship between free Trp concentrations in the shoot tissues and nitrate availability. Values are means ± SD of three repeats of 18-20 root cells and shoot palisade cells provided by 6 seedlings.

## Slide 5
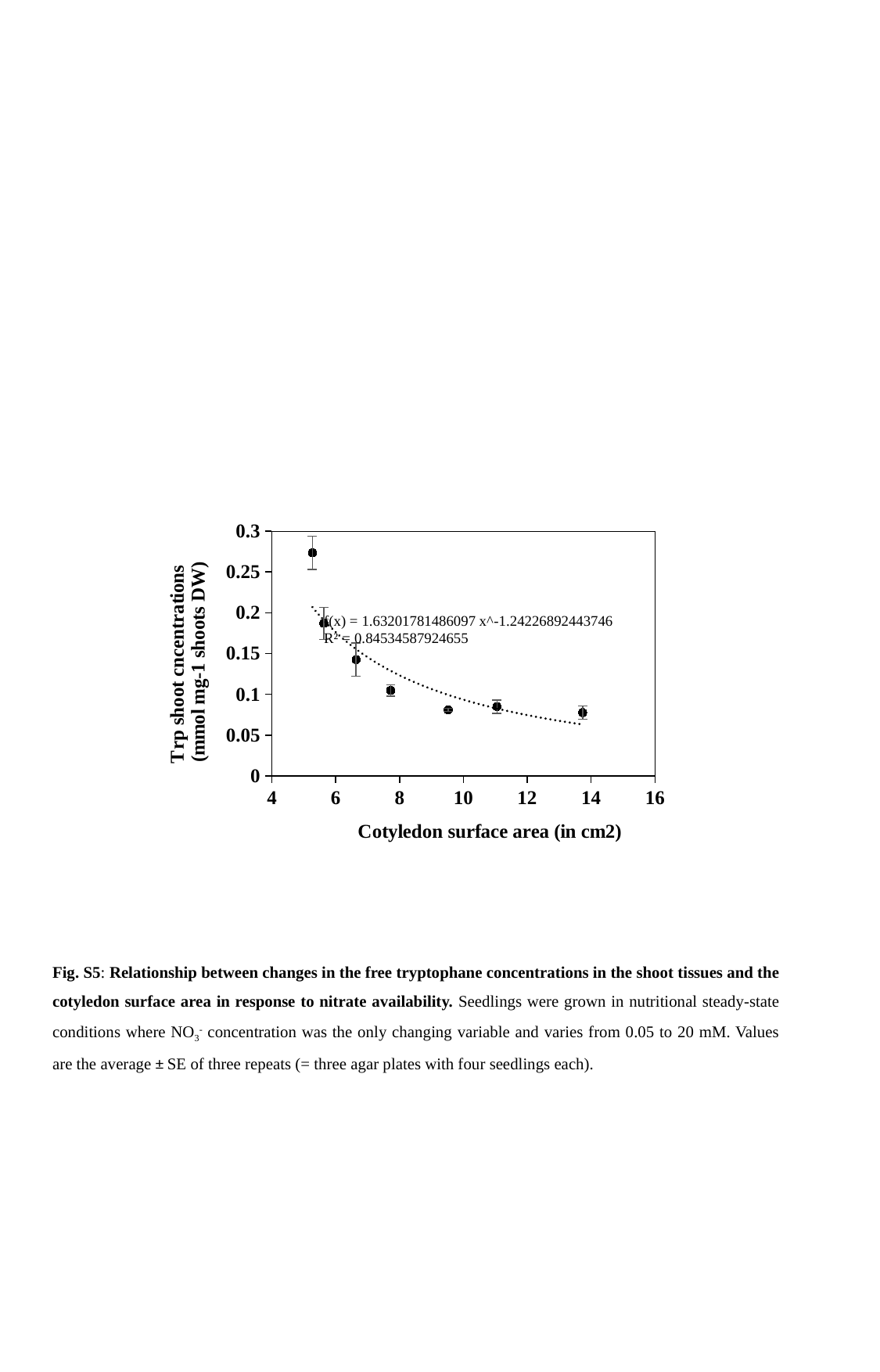

### Chart
| Category | |
|---|---|Fig. S5: Relationship between changes in the free tryptophane concentrations in the shoot tissues and the cotyledon surface area in response to nitrate availability. Seedlings were grown in nutritional steady-state conditions where NO3- concentration was the only changing variable and varies from 0.05 to 20 mM. Values are the average ± SE of three repeats (= three agar plates with four seedlings each).

## Slide 6
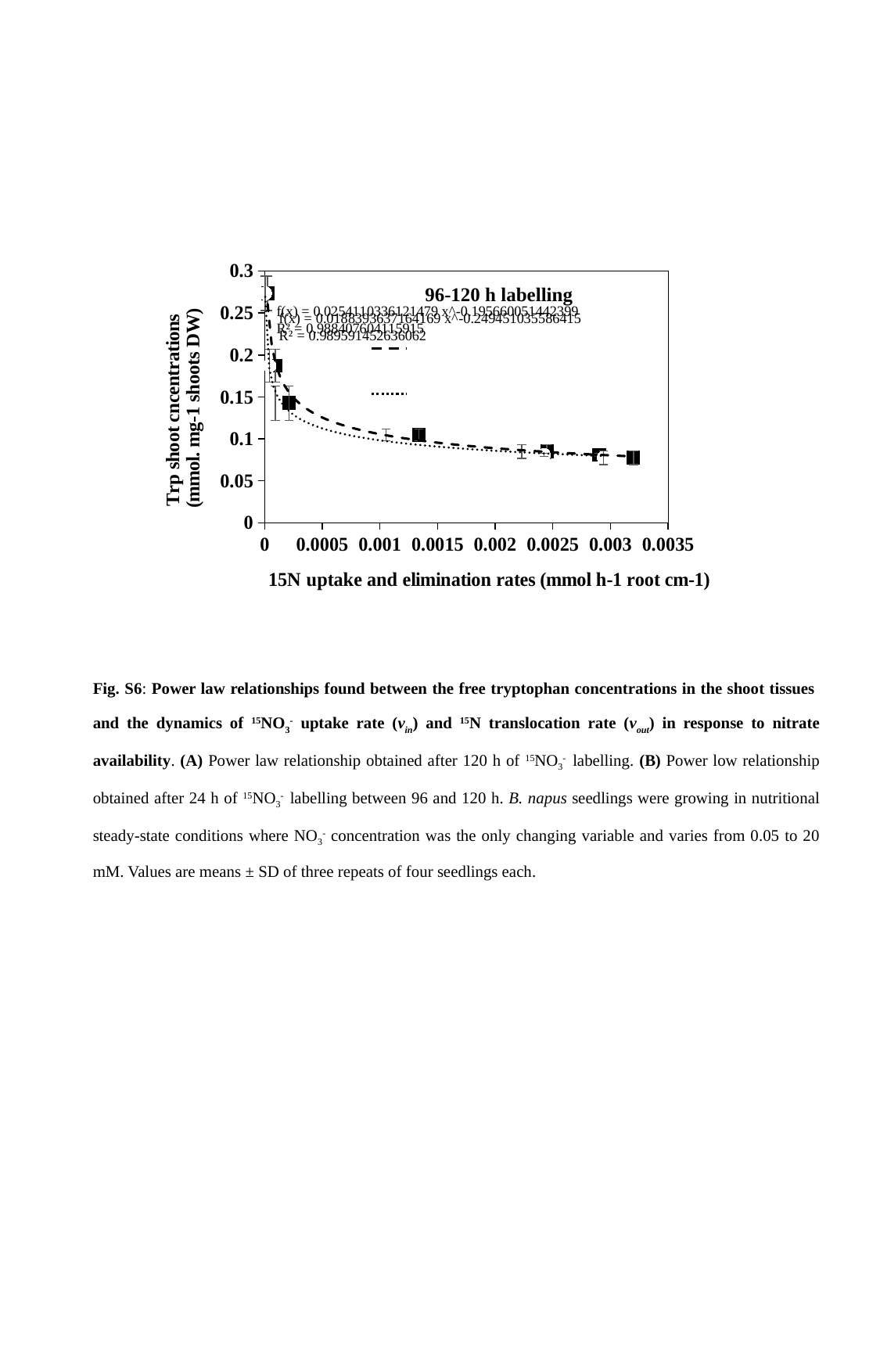

### Chart
| Category | | |
|---|---|---|96-120 h labelling
Fig. S6: Power law relationships found between the free tryptophan concentrations in the shoot tissues and the dynamics of 15NO3- uptake rate (vin) and 15N translocation rate (vout) in response to nitrate availability. (A) Power law relationship obtained after 120 h of 15NO3- labelling. (B) Power low relationship obtained after 24 h of 15NO3- labelling between 96 and 120 h. B. napus seedlings were growing in nutritional steady-state conditions where NO3- concentration was the only changing variable and varies from 0.05 to 20 mM. Values are means ± SD of three repeats of four seedlings each.

## Slide 7
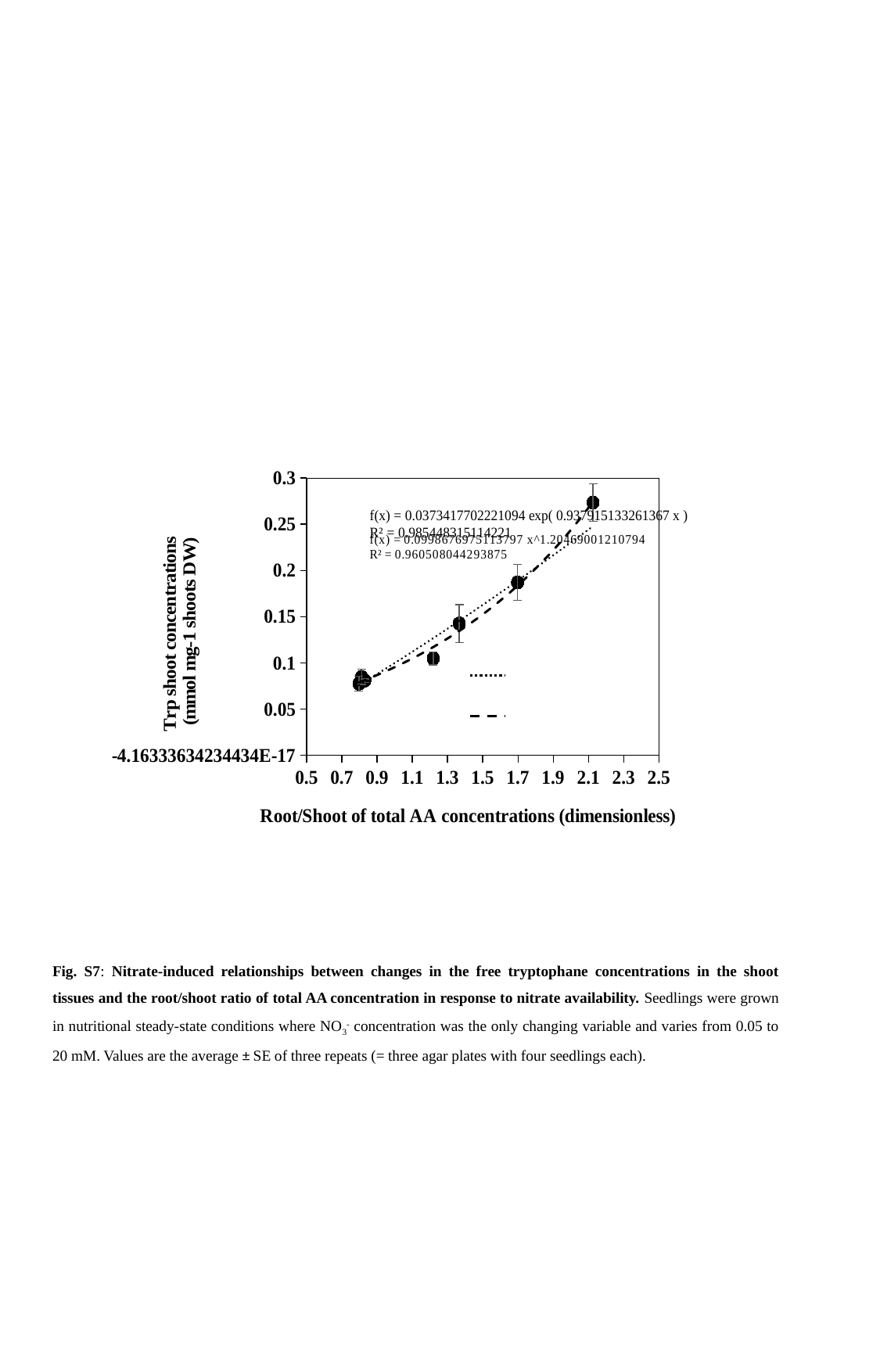

### Chart
| Category | |
|---|---|Fig. S7: Nitrate-induced relationships between changes in the free tryptophane concentrations in the shoot tissues and the root/shoot ratio of total AA concentration in response to nitrate availability. Seedlings were grown in nutritional steady-state conditions where NO3- concentration was the only changing variable and varies from 0.05 to 20 mM. Values are the average ± SE of three repeats (= three agar plates with four seedlings each).
