# Supplementary material for: Combined Allosteric Responses Explain the Bifurcation in Non-Linear Dynamics of 15N Root Fluxes Under Nutritional Steady-State Conditions for Nitrate
Source: Front Plant Sci. 2020 Aug 28;11:1253. doi: 10.3389/fpls.2020.01253 (PMC7770280; doi:10.3389/fpls.2020.01253)
Supplement: Supplementary file 2 [file Table_1.docx]

**Table SI:   Ratio of the velocities *v_out_ /v_out_* of *^15^N fluxes* during the light/darkness cycle.** Fifteen-day-old seedlings, cultivated in a nutrient solution with 1 mM KNO_3_ for 1 week in culture room, were acclimated for 1.5 h in a nutrient solution containing either 100 μM or 5 mM KNO_3_. ^15^NO_3_^-^ influx rates were then determined at 100 μM and 5 mM for 5 minutes with labelling solution of K^15^NO_3_ (atom % ^15^N: 99) in a time schedule of 3, 6, 9, 12 and 15 h after the onset of light (*i.e.*16 h light) and after the onset of darkness period in a time schedule of 0, 2, 4, 6 and 8 h (*i.e.* 8 h darkness). The thermoperiod was 20°C (day) and 15°C (night) and PAR of 300 μmol m^-2^ s^-1^.

| **KNO_3_**  **Treatments** | **Photoperiod**  **duration** | ***V_in_***  *_μmol_ ^15^_N h_^-1^_root DW_^-1^* | ***V_app_***  *_μmol_ ^15^_N h_^-1^_root DW_^-1^* | ***V_out_***  *_μmol_ ^15^_N h_^-1^_root DW_^-1^* | ***V_out_ / V_app_***  *_dimensionless_* |
| --- | --- | --- | --- | --- | --- |
| *0.1 mM* | **Light**  16 h | 62.5 ± 11.2 | 31 ± 0.6 | 31.5 ± 6.8 | **1.04** ± 0.21  N =10 |
|  | **Darkness**  8 h | 91.3 ± 8.8 | 48.9 ± 5.7 | 42.5 ± 12.3 | **1.26** ± 0.45  N = 8 |
| *5 mM* | **Light**  16 h | 180 ± 25.3 | 110 ± 19.2 | 72 ± 15.5 | **1.61** ± 0.39  N =10 |
|  | **Darkness**  8 h | 237.5 ± 47.2 | 156.9 ± 46.48 | 80.6 ± 23.2 | **2.15** ± 0.47  N = 8 |
